# Estrogen-dependent activation of NCOA3 couples with p300 and NF-κB to mediate antiapoptotic genes in ER-positive breast cancer cells

**DOI:** 10.1007/s12672-023-00635-0

**Published:** 2023-02-28

**Authors:** Jun Wang, Zhiyong Zhou

**Affiliations:** 1 Department of Breast Surgery, Jiangxi Provincial People’s Hospital, The First Affiliated Hospital of Nanchang Medical College, Nanchang, 330006 Jiangxi China; 2 Department of Oncology, Jiangxi Provincial People’s Hospital, The First Affiliated Hospital of Nanchang Medical College, 92 Aiguo Rd, Donghu District, Nanchang, 330006 Jiangxi China

**Keywords:** ER-positive breast cancer, NCOA3, p300, NF-κB, Antiapoptotic gene

## Abstract

Circumvention of apoptosis by the elevation of antiapoptotic proteins is an important cause of carcinogenesis. The induction of antiapoptotic genes, including B-cell CLL/lymphoma 2 (*BCL2*), BCL2 related protein A1 (*BCL2A1*), BCL2 like 1 (*BCL2L1*), *BCL2L2*, and myeloid cell leukemia 1 (*MCL1*), has been observed in multiple cancers, including breast cancer. However, the underlying mechanisms of their overexpression are still being investigated. Here, we revealed that *BCL2*, *BCL2A1*, *BCL2L2*, and *MCL1* but not *BCL2L1* were overexpressed in estrogen receptor (ER)-positive breast cancer cells and clinical biopsies. Stimulation with estrogen in ER-positive cell lines resulted in a dose-dependent increase in *BCL2*, *BCL2A1*, *BCL2L2*, and *MCL1* mRNA levels. Molecular investigation revealed that nuclear factor kappa B (NF-κB) recruited histone acetyltransferase p300 and nuclear receptor coactivator 3 (NCOA3) to form a transcriptional complex. This complex docked the promoters of *BCL2*, *BCL2A1*, *BCL2L2*, and *MCL1* and activated their expression. Interestingly, estrogen exposure dose-dependently activated NCOA3. Depletion of the NCOA3-p300-NF-κB components or blockage of NCOA3 function with inhibitors (gossypol and bufalin) in ER-positive cells suppressed *BCL2*, *BCL2A1*, *BCL2L2*, and *MCL1* expression, while also decreasing cell viability, colony formation, cell invasion, and tumor growth. Collectively, our results demonstrate an upstream signaling that activates four antiapoptotic genes in ER-positive breast cancer cells. Importantly, our results also imply that targeting NCOA3 or blocking the assembly of the NCOA3-p300-NF-κB complex may be promising therapeutic strategies for treating ER-positive breast cancer.

**Supplementary Information:**

The online version contains supplementary material available at 10.1007/s12672-023-00635-0.

## Introduction

Breast cancer is an important cause of cancer-related deaths in women [1, 2], with an estimated incidence rate of 129.1/100,000 women and a death rate of 19.9/100,000 each year [1, 2]. Based on the genes expressed by tumor cells, breast cancers are divided into five subtypes: luminal A breast cancer [estrogen receptor (ER)-positive, progesterone receptor (PR)-positive, human epidermal growth factor receptor 2 (HER2)-negative, and low levels of Ki-67], luminal B breast cancer (ER-positive, HER2-negative, and high levels of Ki-67), luminal B-like breast cancer (ER-positive and HER2 positive), HER2-enriched breast cancer (ER-negative, PR-negative, and HER2-positive), and triple-negative breast cancer (ER-negative, PR-negative, and HER2-negative) [3, 4]. ER-positive patients account for approximately 60–70% of all breast cancer cases, and ER overexpression is significantly associated with poor prognosis [3, 4].

Breast tumorigenesis can occur due to dysregulation of proapoptotic and antiapoptotic signaling, and this dysregulation also increases acquired resistance during therapy [5, 6]. As in other types of cancer cells, intrinsic apoptosis in breast cancer cells is mainly controlled by two subgroups of B-cell CLL/lymphoma 2 (Bcl-2) proteins, consisting of 5 antiapoptotic proteins [Bcl2 related protein 1 (Bcl2-A1), Bcl-2, Bcl2 like 1 (Bcl2-L1, also known as Bcl-xL), Bcl2 like 2 (Bcl2-L2, also known as Bcl-w) and myeloid cell leukemia 1 (Mcl-1)] and 9 proapoptotic proteins [Bcl-2 associated agonist of cell death [Bad], Bcl-2 associated X (Bax), Bak1 (Bcl-2 antagonist/killer 1), BH3 interacting domain death agonist (Bid), Bcl-2 interacting killer (Bik), Bcl-2 interacting mediator of cell death (Bim), harakiri (Hrk), phorbol-12-myristate-13-acetate-induced protein 1 (PMAIP1, also known as Noxa), and p53 upregulated modulator of apoptosis (Puma)] [7, 8]. The five antiapoptotic genes (*BCL2*, *BCL2A1*, *BCL2L1*, *BCL2L2,* and *MCL1*) are frequently overexpressed in ER-positive breast cancer cells, and these genes have been used as clinical biomarkers in the diagnosis of breast cancer [9–12]. In a study that recruited 11,212 women, Dawson and colleagues identified BCL2 as an independent indicator for all types of early-stage breast tumors, not just ER-positive tumors [13].

ER signaling mediates gene expression through classical and non-classical pathways [14]. In the classical pathway, β-estradiol (also called E2) binds to the ER, causing the conformational change of the ER and enabling its release from the heat shock protein complex [14]. The ER translocates from the cytoplasm to the nucleus, where it coordinates with transcriptional coactivators or corepressors to regulate gene expression by binding to the estrogen response elements (ERE) [14]. Moreover, the ER can also mediate gene expression by coordinating with different transcription factors, such as Activating Protein 1 (AP1), Specificity Protein 1 (SP1), and Nuclear Factor Kappa B (NF-κB) [14]. Among the five antiapoptotic genes, three (*BCL2, BCL2A1,* and *MCL1*) can be regulated by NF-κB in different types of cancer cells [15–17].

NF-κB consists of five subunit proteins, p65 (RelA), RelB, c-Rel, p50 (NF-κB1), and p52 (NF-κB2), which can form homo- and heterodimers to regulate gene expression [15–17]. Whether NF-κB can also mediate the expression of the five antiapoptotic genes in breast cancer cells is unclear. Moreover, a study has shown that *BCL2* mRNA and protein levels are upregulated, while *BCL2L1* mRNA and protein levels are downregulated, by E2 in the ER-positive MCF7 cell line. These findings suggest a substantial complexity regarding the regulation of antiapoptotic genes [18].

Three nuclear receptor coactivators (NCOAs), namely NCOA1, NCOA2, and NCOA3, which are frequently overexpressed in breast cancer cells and are involved in estrogen-mediated cancer cell proliferation [19, 20]. However, the NCOA-associated transcriptional complexes and their target genes remain unknown.

The observed interaction between ER responses and apoptosis strongly suggests that a reasonable therapeutic treatment strategy for breast cancer would be pharmacological inhibition of antiapoptotic proteins [5–8]. However, pharmacological inhibition of Bcl-2 and Bcl-xL, alone or combination, in breast cancer cells causes the induction of Mcl1 and therefore fails to inhibit tumor cell growth [5–8]. In the current study, we revealed a transcriptional mechanism by which NF-κB recruits a histone acetyltransferase p300 and the estrogen-activated NCOA3 to induce the expression of *BCL2*, *BCL2A1*, *BCL2L2*, and *MCL1,* but not *BCL2L1*. Knockdown or pharmacological inhibition of NCOA3 significantly suppressed the expression of *BCL2*, *BCL2A1*, *BCL2L2*, and *MCL1*, suggesting that targeting NCOA3 or disrupting the NCOA3-p300-NF-κB complex may represent new therapeutic strategies for treating breast cancer.

## Materials and methods

### Cells and cell culture

Four human ER-positive breast cancer cell lines (MCF-7, T47D, BT474, and ZR-751), four ER-negative cell lines (SKBR3, MDAMB453, BT549, and HCC70), and one normal human breast epithelial cell line (HME1) were purchased from the American Type Culture Collection (ATCC) (Manassas, VA, USA). The other normal human breast epithelial cell line (HMEC2.6) was obtained from Applied Biological Materials (ABM) (Richmond, BC, Canada; #T0454). All cell lines were cultured in Dulbecco’s Modified Eagle Medium (DMEM) containing 10% charcoal-stripped fetal bovine serum (FBS) (Thermo Fisher, Shanghai, China; #12676029) and 50 µg/mL penicillin–streptomycin (Thermo Fisher; #15140122). Cells were grown in a 37 °C humidified chamber in a 5% CO_2_ atmosphere, with medium changes twice per week.

### Collection of human breast tumor biopsies

The protocol (#BC2015-013) for human breast tumor biopsy collection was approved by the Ethics Board of Jiangxi Provincial People’s Hospital. All enrolled patients provided written informed consent. In total, 73 ER-negative and 102 ER-positive breast tumors and their adjacent normal tissues were collected. All patients were diagnosed and underwent surgery from 2015 to 2019 in the Department of Oncology, Jiangxi Provincial People’s Hospital, Nanchang, China. The basic information (ages and tumor stages) of these patients are provided in Table S1.

### Vector construction and plasmid purification

Full-length coding sequences of RelA/p65, NFKB1/p50, p300, and NCOA3 were amplified by the polymerase chain reaction (PCR) with the Phusion High-Fidelity DNA Polymerase (New England Biolabs, Shanghai, China; #M0530S). The primers are listed in Table S2. The purified DNA samples were digested with EcoRI + XhoI (insertion into pCDNA3-Flag empty vector) and BamHI + NotI (insertion into pCDNA3-Myc empty vector) and then ligated into vectors. The transformants were selected on ampicillin-resistant agar plates, and bacterial colonies were validated using PCR and DNA sequencing. Plasmids were isolated using the GenElute Plasmid Miniprep Kit (Sigma-Aldrich, Shanghai, China; #PLN70).

### Cell transfection

For gene knockdown (KD), a pLKO.1-puro non-target shRNA control (for the generation of Control-KD cell lines) and two specific short hairpin RNAs (shRNAs) (Table S3) targeting each gene were individually mixed with transfection medium (Santa Cruz Biotechnology, Shanghai, China; #sc-108062) and then transfected into HME1, T47D, and BT549 cells. The transfected cells were incubated in DMEM at 37 °C for 7 h, followed by selection in DMEM containing 2 μg/mL puromycin (Thermo Fisher; #A1113802) for 48 h. Single puromycin-resistant cells were collected and expanded. Two independent clones of Control-KD (1 and 2) and two independent KD clones of each gene, namely RelA-KD1/2, NFKB1-KD1/2, NCOA3-KD1/2, and p300-KD1/2, were selected for the experiments. For gene overexpression, Flag-tagged and Myc-tagged vectors (Table S2) were transfected using Lipofectamine 3000 (Thermo Fisher; #L3000015) and culturing at 37 °C for 48 h.

### Cell treatments

Cells were grown in DMEM containing 10% charcoal-stripped FBS until 80% confluence, followed by treatments with phosphate buffered saline (PBS; control) or 5 or 10 nM β-estradiol (E2; Sigma-Aldrich; #E1024) in PBS for 4 h. For E2 treatment in the MCF7 cell line, both PBS and E2-PBS buffers contained 20 nM MLN4924 (a small molecule inhibitor of the Nedd8 activating enzyme) (Sigma-Aldrich; # 5054770001). After two washes with ice-cold PBS, the cells were used for RNA and protein isolation. The T47D cells were also cultured in DMEM containing 10% charcoal-stripped FBS and then treated with PBS, 100 nM bufalin (Sigma-Aldrich; #B0261) in PBS, or 5 μM gossypol (Sigma-Aldrich; #PHL83856) in PBS for 6 h, followed by additional treatment with or without 10 nM E2 for another 4 h. The treated cells were washed twice with ice-cold PBS buffer and used for RNA and protein isolation.

### Total RNA isolation and quantitative real-time PCR (RT-qPCR)

After aspiration of the culture medium, approximately 5 × 10^6^ cells were washed twice with ice-cold phosphate-buffered saline (PBS) (pH7.4) and lysed in 1 mL TRIzol (Thermo Fisher; 15596018) for 10 min at 4 °C. Chloroform (250 µL) (Thermo Fisher; C4920115) was added and the tubes were shaken vigorously for about 15 s, then held at 4 °C for 15 min. The resulting cell extracts were centrifuged at 13,000*g* for 10 min, and the supernatants were mixed with 550 µL isopropanol (Thermo Fisher; #P750317) to precipitate the RNA. The tubes were kept at − 20 °C overnight, followed by centrifugation at 15,000*g* for 20 min. The extracted RNA was dissolved in 50 µL RNAse-free ddH_2_O and its concentration was determined using a Nanodrop instrument.

For each sample, 1 µg total RNA was used to synthesize first-strand cDNA with the SuperScript III First-Strand Synthesis System (Thermo Fisher; #18080051). The obtained cDNA was diluted 20-fold, and the gene expression levels were determined by RT-qPCR analysis using the SYBR GreenER qPCR SuperMix Universal Kit (Thermo Fisher; #1176202 K) with the primers listed in Table S4. The relative expression levels of the individual genes were quantified using the 2^−ΔΔ*CT*^ method and β-actin as an internal control.

### Western blotting analysis

Cells (approximately 5 × 10^6^) were lysed in 200 µL RIPA Lysis and Extraction Buffer (Thermo Fisher; #89900), and the tubes were kept on ice for 20 min. The resulting cell lysates were centrifuged at 13,000*g* for 20 min, and the total protein levels in the supernatants were quantified using a Nanodrop instrument. Equal amounts of protein were separated by electrophoresis on 10% SDS-PAGE gels and then transferred to polyvinylidene fluoride (PVDF) membranes (Thermo Fisher; #88520) and blocked with 5% fat-free milk for 1 h. The membranes were then probed with primary antibodies, including anti-Bcl2 (Abcam, Shanghai, China; #ab241548), anti-Bcl2A1 (Thermo Fisher; #TA806463), anti-Bcl-xL (Abcam; #ab32370), anti-Bcl-w (Thermo Fisher; #MA515076), anti-Mcl1 (Abcam; #ab246684), anti-p65 (Abcam; #ab32536), anti-p50 (Abcam; #ab283688), anti-p300 (Abcam; #ab10485), anti-NCOA3 (Abcam; #ab133611), anti-Flag (Abcam; #ab49763), anti-Myc (Abcam; #ab32), anti-ERα (Sigma-Aldrich; #SAB4500813), anti-ERβ (Abcam; #ab3576), anti-GAPDH (glyceraldehyde 3-phosphate dehydrogenase) (Abcam; #ab8245), anti-Bax (Bcl-2 associated X-protein) (Abcam; #ab270742), anti-Bak (Bcl-2 homologous antagonist/killer; Abcam; #ab32371), and anti-caspase 9 (Cell Signaling, Shanghai, China; #9502). After washing 5 times with PBS containing Tween20 (PBST), the membranes were probed with secondary antibodies, including anti-mouse IgG H&L (Abcam; #ab205719) and anti-Rabbit IgG H&L (Abcam; #ab205718). Protein bands were detected using the Pierce™ ECL Western Blotting Substrate Kit (Thermo Fisher; #32109). Protein band intensity was normalized using ImageJ software (Fiji version 1.44a).

### Cell viability, invasion, and colony formation assays

Cells were seeded in a 96-well plate at a density of 10^4^ cells/well in 100 µL of DMEM containing 10% charcoal-stripped FBS. Cell viability was determined using a 3-(4,5-dimethylthiazol-2-yl)-2,5-diphenyl-2H-tetrazolium bromide (MTT) assay kit (Thermo Fisher; V13154) at different time points (0, 1, 2, 3, 4, and 5 days). For cell invasion assays, 1 × 10^5^ cells/well were seeded in the upper compartment of Nunc polycarbonate cell culture inserts (Thermo Fisher; #140644) coated with extracellular matrix (Thermo Fisher; #33010018**).** After incubation at 37 °C for 48 h, invading cells in the lower insert were fixed with 5% glutaraldehyde (Thermo Fisher; #A17876) at 37 °C for 10 min, followed by staining with 0.1% crystal violet (Thermo Fisher; #B2193236) in 2% ethanol for an additional 20 min at 37 °C. The cells were counted after washing off excess dye with ddH_2_O.

For the colony formation assay, cells were seeded into 6-well plates (Thermo Fisher; #145380) at a density of 1 × 10^3^ cells/well and cultured in 2 mL DMEM containing 10% charcoal-stripped FBS for three weeks. Cell colonies were fixed with 5% glutaraldehyde at 37 °C for 10 min, followed by staining with 0.1% crystal violet and rinsing with ddH_2_O to remove excess dye. Colony numbers were counted using ImageJ software.

### Tumor xenograft model

Animal experiments were performed following a protocol (NCU202019BM) reviewed and approved by the Institutional Animal Care and Use Committee of Jiangxi Provincial People’s Hospital. The in vivo effects of depletion of NCOA3-p300-NF-κB members were determined by mixing 5.0 × 10^5^ Control-KD1, RelA-KD1, NFKB1-KD1, p300-KD1, and NCOA3-KD1 cells (all in T47D background) in 100 μL of PBS with 50% Matrigel (Sigma-Aldrich; #CLS354234) and inoculating them subcutaneously into one armpit of female BALB/c nude mice (22–25 g) (n = 10 for each group) (Vital River Laboratories, Beijing, China). Each group of mice was randomly divided into two subgroups. One mouse subgroup was implanted with a 0.18 mg E2 pellet (Innovative Research Of America, Sarasota, FL, USA; #NC1775204) following a previous protocol [21]. The other group underwent the same surgery but without E2 pellet implantation. The position of the E2 pellet implantation was contralateral to the cell injection site between the skin and the peritoneal wall. Tumor sizes were measured every 5 days with a digital caliper (Thermo Fisher; #0666416), and tumor volumes were calculated using the formula: volumes = ½ (Length × Width^2^). For evaluation of the in vivo effects of NCOA3 inhibitors, T47D cells (5.0 × 10^5^) were injected into female BALB/c nude mice (n = 60), and half of the mice were further implanted with a 0.18 mg E2 pellet. After the tumor volumes reached approximately 150 mm^3^, the mice were randomly grouped and injected intraperitoneally every 5 days with 1.5 mg/kg bufalin (n = 10), 50 mg/kg gossypol (n = 10), or PBS (control, n = 10). Tumor sizes were measured with a digital caliper every 5 days.

### Immunoprecipitation and mass spectrometry analysis

Cells (approximately 1 × 10^7^) were lysed in 400 µL RIPA Lysis and Extraction Buffer supplemented with 1 × protease inhibitor cocktail (Sigma-Aldrich; #P1860). Cell extracts were centrifuged at 13,000*g* for 20 min, and the supernatants were immunoprecipitated using anti-p65-coupled protein A agarose (Thermo Fisher; #20333) and IgG-coupled protein A agarose. The purified p65-associated protein complex was used for mass spectrometry analysis to identify p65-interacting proteins, following a previously reported protocol [22].

### Co-immunoprecipitation (Co-IP) assay

Different combinations of plasmids, including pCDNA3-Flag-EV (empty vector) + pCDNA3-Myc-EV, pCDNA3-Flag-EV + pCDNA3-Myc-p50, pCDNA3-Flag-EV + pCDNA3-Myc-p300, pCDNA3-Flag-EV + pCDNA3-Myc-NCOA3, pCDNA3-Flag-p65 + pCDNA3-Myc-EV, pCDNA3-Flag-p65 + pCDNA3-Myc-p50, pCDNA3-Flag-p65 + pCDNA3-Myc-p300, pCDNA3-Flag-p65 + pCDNA3-Myc-NCOA3, pCDNA3-Flag-EV + pCDNA3-Myc-p65, pCDNA3-Flag-NCOA3 + pCDNA3-Myc-EV, pCDNA3-Flag-NCOA3 + pCDNA3-Myc-p50, pCDNA3-Flag-NCOA3 + pCDNA3-Myc-p65, and pCDNA3-Flag-NCOA3 + pCDNA3-Myc-p300, were cotransfected into T47D cells and incubated at 37 °C for 48 h. The cells were lysed in 200 µL RIPA Lysis and Extraction Buffer supplemented with 1 × protease inhibitor cocktail. After centrifuging at 13,000*g* for 20 min, 20 µL of the supernatants were removed for use as input proteins. The remaining 180 µL was immunoprecipitated using anti-Flag agarose (Abcam; #ab270704). The input and output proteins were probed with anti-Flag and anti-Myc antibodies.

### Chromatin immunoprecipitation (ChIP) assay

Cells (approximately 4 × 10^7^) at 80% confluence were treated with or without 10 nM E2 for 4 h. After rinsing twice with ice-cold PBS, the cells were subjected to ChIP assay procedures following a previously published protocol [23], and the cell lysates were immunoprecipitated using different antibodies (anti-p65, anti-p50, anti-p300, and anti-NCOA3) linked to protein G agarose (Thermo Fisher; #20397). The purified input and output DNAs were used for RT-qPCR analysis with the primers shown in Table S5. The enrichment of p65, p50, p300, and NCOA3 on the promoters of *BCL2*, BCL2A1, *BCL2L1*, *BCL2L2*, and *MCL1* was quantified using the method of % input = 2^−ΔΔCT^, in which ΔCT = Ct^output^−Ct^input^.

### Statistical analysis

All experiments in this study were independently repeated in triplicate, and the data were presented as the mean values ± standard deviation (SD). All analyses of differences between groups were performed using one-way ANOVA coupled with Tukey's post hoc test and the SPSS (Statistical Package for the Social Sciences) statistics software (IBM, NY, USA; version 20). *P* < 0.05 was considered statistically significant.

## Results

### The expression levels of *BCL2*, *BCL2A1*, *BCL2L2*, and *MCL1* were increased in ER-positive cell lines and clinical biopsies

**Fig. 1:**
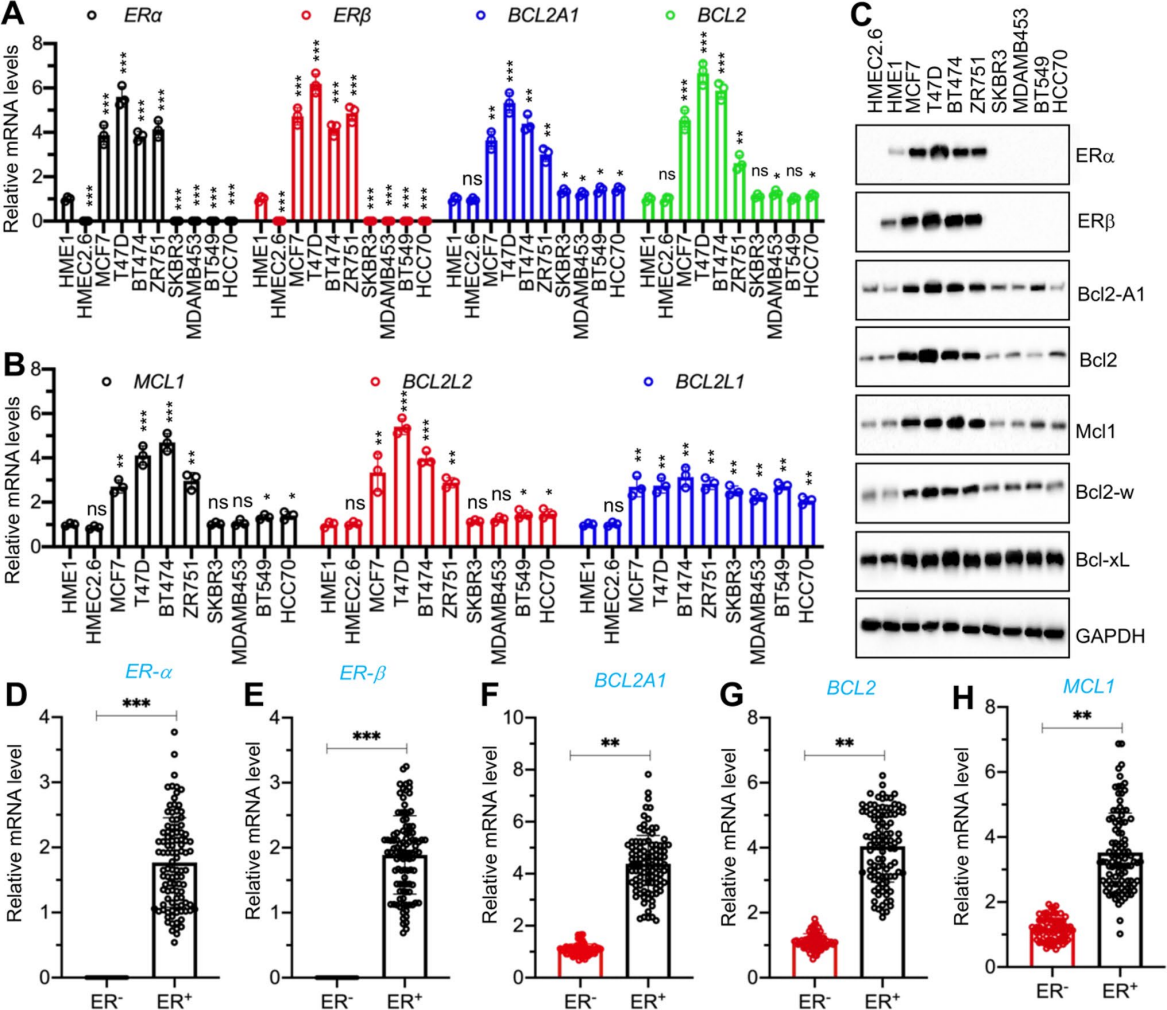
Antiapoptotic genes and their encoded protein levels in ER-positive and ER-negative cells. **A** and **B** The mRNA levels of *ERα*, *ERβ*, and antiapoptotic genes in breast cancer cell lines**.** Total RNA samples from HME1, HMEC2.6, MCF7, T47D, BT474, ZR751, SKBR3, MDAMB453, BT549, and HCC70 were used for RT-qPCR analyses to measure mRNA expression. **A** *ERα*, *ERβ*, *BCL2A1*, and *BCL2*. **B** *MCL1*, *BCL2L2*, and *BCL2L1*. Three independent replicates (n = 3 for each replicate) were performed, and the results represent means of three replicates ± SD. **C** The protein levels of ER and antiapoptotic proteins. Total protein extracts from cells in (A) were used for immunoblots to measure the protein levels of ERα, ERβ, Bcl2A1, Bcl2, Mcl1, Bcl-w, Bcl-xL, and GAPDH (loading control). Three independent replicates were performed. For each lane in a replicate, three independent protein samples were mixed at equal weights (20 μg for each). One representative group of immunoblot images is shown. **D**–**H** The mRNA levels of *ERα*, *ERβ*, and antiapoptotic genes in ER-negative and ER-positive tumor samples. RNA samples from ER-negative (n = 73) and ER-positive (n = 102) tumor samples were used for RT-qPCR analyses to measure mRNA levels of *ERα *(**D**), *ERβ* (**E**), *BCL2A1* (**F**), *BCL2* (**G**), and *MCL1* (**H**), Three independent replicates were performed, and one group of representative results is shown. Significant differences were determined by one-way ANOVA, followed by Tukey's post hoc test. * *P* < 0.05, ** *P* < 0.01, and *** *P* < 0.001

We determined whether the expression levels of five antiapoptotic genes were different in ER-positive and ER-negative cell lines by examining two normal breast epithelial cell lines (HMEC2.6 and HME1), four ER-positive breast cancer cell lines (MCF-7, T47D, BT474, and ZR-751), and four ER-negative cell lines (SKBR3, MDAMB-453, BT549, and HCC70). The mRNA expression levels of two ER members (*ERα* and *ERβ*) were much lower in the HME1 cells than in the four ER-positive cell lines (Fig. 1A). By contrast, HMEC2.6, SKBR3, MDAMB-453, BT549, and HCC70 were all ER-negative cell lines. Using the same RNA samples, we observed a significant induction of *BCL2*, *BCL2A1*, *BCL2L2*, and *MCL1* genes in all four ER-positive cancer cell lines compared to the breast epithelial cell lines (Fig. 1A and B), but only a slight induction in the ER-negative cell lines (Fig. 1A and B).

By contrast, the *BCL2L1* mRNA level was increased in both ER-positive and ER-negative cell lines, but the difference between these two groups of cells was not statistically significant (Fig. 1B). We also consistently observed similar expression patterns for the two ER members and five antiapoptotic protein levels and their mRNA levels in all the examined cell lines (Figs. 1C, S1A, and S1B).

We examined the expression levels of *BCL2*, *BCL2A1*, *BCL2L2*, and *MCL1* in clinical biopsies by collecting 102 ER-positive and 73 ER-negative breast tumor samples (Fig. 1D and E). Similar to the findings in the ER-positive and ER-negative cell lines in Fig. 1A, B, we also observed that *BCL2*, *BCL2A1*, *BCL2L2*, and *MCL1*, but not *BCL2L1*, were highly expressed in ER-positive tumors compared to those in ER-negative tumors (Figs. 1F–G, S1C, and S1D). Pearson’s correlation tests using gene expression levels indicated a positive correlation between the mRNA levels of *BCL2*, *BCL2A1*, *BCL2L2*, and *MCL1,* but not *BCL2L1,* and the mRNA levels of ERα and ERβ (Figures S2A–J). These results suggested that *BCL2*, *BCL2A1*, *BCL2L2*, and *MCL1* might be estrogen-dependent genes.

### The expression levels of *BCL2*, *BCL2A1*, *BCL2L2*, and *MCL1* were induced by E2

We next examined the direct response of *BCL2*, *BCL2A1*, *BCL2L1*, *BCL2L2*, and *MCL1* to estrogen by treating HME1 cells, two ER-positive cell lines (T47D and MCF7), and two ER-negative cell lines (BT549 and HCC70) with 5 or 10 nM E2. The E2 treatments caused dose-dependent inductions of mRNA and protein expression of *BCL2*, *BCL2A1*, *BCL2L2*, and *MCL1,* but not of *BCL2L1,* in the HME1, T47D, and MCF7 cells (Figures S3–S6). However, E2 treatments did not induce significant changes in the mRNA and protein levels of *BCL2*, *BCL2A1*, *BCL2L2*, or *MCL1* in the two ER-negative cell lines (Figures S3–S6). The mRNA and protein levels of *BCL2L1* were not changed following E2 treatments in any of the cell lines (Figures S3–S6). These results suggested that E2 induced the mRNA and protein expression of *BCL2*, *BCL2A1*, *BCL2L2*, and *MCL1,* but not of *BCL2L1*.

### NF-κB controlled the expression of *BCL2*, *BCL2A1*, *BCL2L2*, and *MCL1* at the transcriptional level

**Fig. 2:**
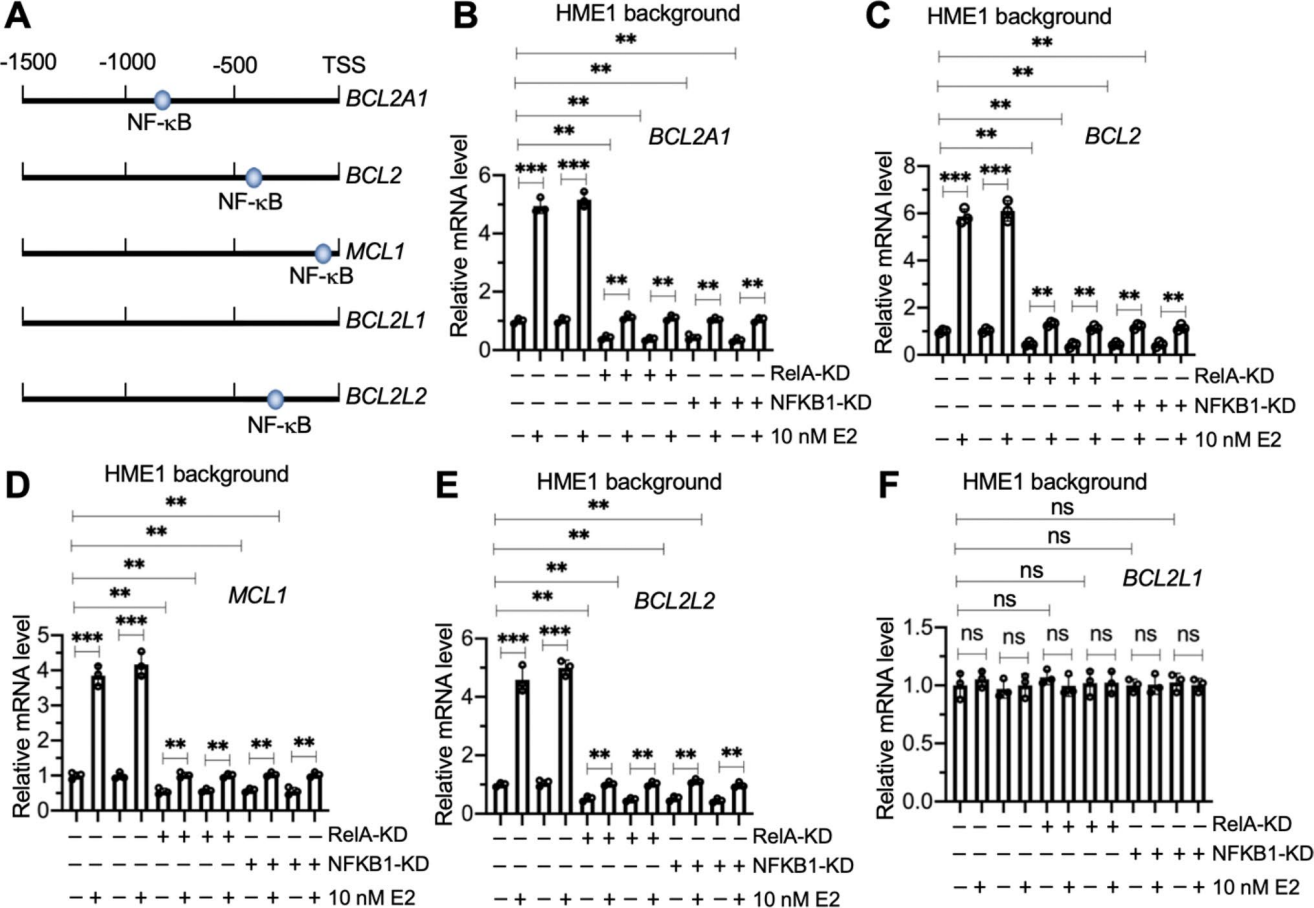
The expression levels of *BCL2A1*, *BCL2*, *MCL1*, and *BCL2L2* were controlled by NF-κB. **A** Schematic diagrams of NF-κB binding sites on the promoters of antiapoptotic genes. The promoters (1500 bp length) of *BCL2A1*, *BCL2*, *MCL1*, *BCL2L1*, and *BCL2L2* were searched for NF-κB binding sites using the consensus DNA sequence of 5′-GGGRNYYYCC-3′. The potential NF-κB binding sites are shown. **B**–**F** The mRNA levels of antiapoptotic genes**.** The Control-KD1/2, RelA-KD1/2, and NFKB1-KD1/2 cells in the HME1 background were treated with 10 nM E2 or without E2 (used PBS) for 4 h, followed by RNA isolation and RT-qPCR analyses to measure mRNA levels of *BCL2A1* (**B**), *BCL2* (**C**), *MCL1* (**D**), *BCL2L2* (**E**), and *BCL2L1* (**F**). Three independent replicates (n = 3 for each replicate) were performed, and the results represent means of three replicates ± SD. Significant differences were determined by one-way ANOVA, followed by Tukey's post hoc test. ** *P* < 0.01 and *** *P* < 0.001

The changes in *BCL2*, *BCL2A1*, *BCL2L2*, and *MCL1* mRNA levels in E2-treated HME1, T47D, and MCF7 cells suggested that these genes might be regulated at the transcriptional level. Several studies have shown that *BCL2, BCL2A1,* and *MCL1* are the target genes of the NF-κB transcription factor [15–17]. We found that the promoters of the *BCL2*, *BCL2A1*, *BCL2L2*, and *MCL1* genes, but not the promoter of the *BCL2L1* gene, contained an NF-κB binding site when we predicted the promoters using the consensus DNA sequence of 5ʹ-GGGRNYYYCC-3ʹ in which R represented a purine, Y represented a pyrimidine, and N represented any nucleotide (Fig. 2A).

We created two independent KD cell lines of two NF-κB subunits (p50/NFKB1 and p65/RelA) in the HME1, T47D, MCF7, BT549, and HCC70 backgrounds (Figures S7 and S8) and treated these cells with 10 nM E2 to evaluate the dependence of *BCL2*, *BCL2A1*, *BCL2L2*, and *MCL1* expression on NF-κB. Depletion of the NF-κB subunits decreased the expression of *BCL2*, *BCL2A1*, *BCL2L2*, and *MCL1* in the HME1, T47D, and MCF7 cells even without E2 treatment (Fig. 2B–F, S9, and S10). By contrast, knockdown of NF-κB subunits did not change the expression of *BCL2L1* in the HME1, T47D, or MCF7 backgrounds (Fig. 2B–F, S9, and S10). E2 treatment only slightly induced the expression of *BCL2*, *BCL2A1*, *BCL2L2*, and *MCL1,* but it did not change the expression of *BCL2L1* in the RelA-KD and NFKB1-KD cells in the HME1, T47D, and MCF7 backgrounds (Fig. 2B–F, S9, and S10).

In the ER-negative cells (BT549 and HCC70), depletion of NF-κB subunits decreased the expression of *BCL2*, *BCL2A1*, *BCL2L2*, and *MCL1,* but not of *BCL2L1* (Figures S11 and S12). E2 treatment did not induce the expression of *BCL2*, *BCL2A1*, *BCL2L2*, or *MCL1* (Figures S11 and S12). We concluded that the expression of *BCL2*, *BCL2A1*, *BCL2L2*, and *MCL1,* but not of *BCL2L1,* was controlled by both NF-κB and estrogen at the transcriptional level.

### NF-κB recruited p300 and NCOA3 to assemble a complex in breast cancer cells

**Fig. 3:**
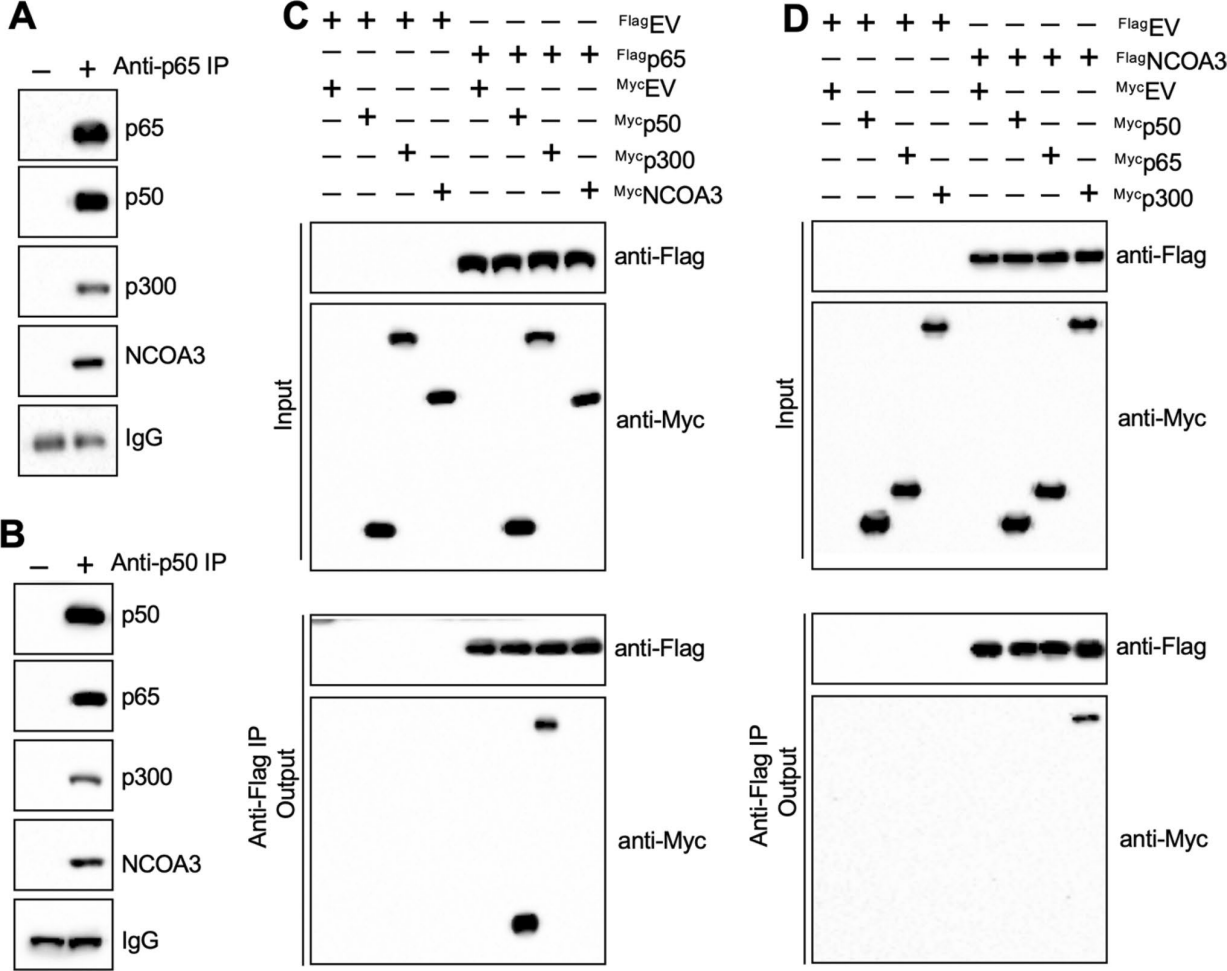
NCOA3 formed a transcriptional complex with p300 and two NF-κB subunits. **A** Immunoprecipitation with p65 pulled down p50, p300, and NCOA3. Cell extracts from T47D cells were immunoprecipitated using anti-p65-conjugated agarose or IgG-conjugated agarose. The purified protein complex was detected using anti-p65, anti-p50, anti-p300, and anti-NCOA3. **B** p50 immunoprecipitated p65, p300, and NCOA3. The same cell extracts as in (**A**) were immunoprecipitated using anti-p50-conjugated agarose or IgG-conjugated agarose. The purified protein complex was detected using anti-p50, anti-p65, anti-p300, and anti-NCOA3. **C** and **D** Co-IP results. T47D cells co-expressing different combinations of Flag-tagged and Myc-tagged plasmids were lysed and immunoprecipitated with anti-Flag- and anti-Myc-agarose. The input and output proteins were probed using anti-Flag and anti-Myc. **C** p65 directly interacted with p50 and p300. **D** NCOA3 directly interacted with p300. For all experiments in this figure, three independent replicates were performed. For each lane in a replicate, three independent protein samples were mixed with equal weights (20 μg for each). One representative group of immunoblot images is shown

We examined how NF-κB might regulate the expression of *BCL2*, *BCL2A1*, *BCL2L2*, and *MCL1* by immunoprecipitation experiments. We immunoprecipitated NF-κB-associated proteins in T47D cell extracts using anti-p65-coupled protein A agarose and analyzing the p65-interacting proteins by mass spectrometry. Among the candidate p65-interacting proteins (Table S6), we identified three known transcriptional regulators: an NF-κB subunit p50, histone acetyltransferase p300, and transcriptional coactivator NCOA3. Using the same immunoprecipitated products as analyzed by mass spectrometry, we verified that p65 could pull down p50, p300, and NCOA3 (Fig. 3A). A further immunoprecipitation experiment in MCF-7 cell extracts using anti-p50-coupled protein A agarose and subsequent western blotting confirmed that p50 also immunoprecipitated p65, p300, and NCOA3 (Fig. 3B).

We also co-expressed Flag-p65 + Myc-p50, Flag-p65 + Myc-p300, Flag-p65 + Myc-NCOA3, Flag-NCOA3 + Myc-p65, Flag-NCOA3 + Myc-p50, and Flag-NCOA3 + Myc-p300 in T47D cells and then performed Co-IP assays. We found that p65 could directly interact with p50 and p300, rather than with NCOA3 (Fig. 3C), and that NCOA3 directly interacted with p300 but not with p65 or p50 (Fig. 3D). These results suggested that a p65/p50 heterodimer recruited p300 and that p300 then bound NCOA3 to assemble a complex.

### E2 treatment induced the expression of *NCOA3* and promoted the assembly of the NCOA3-p300-NF-κB complex

**Fig. 4:**
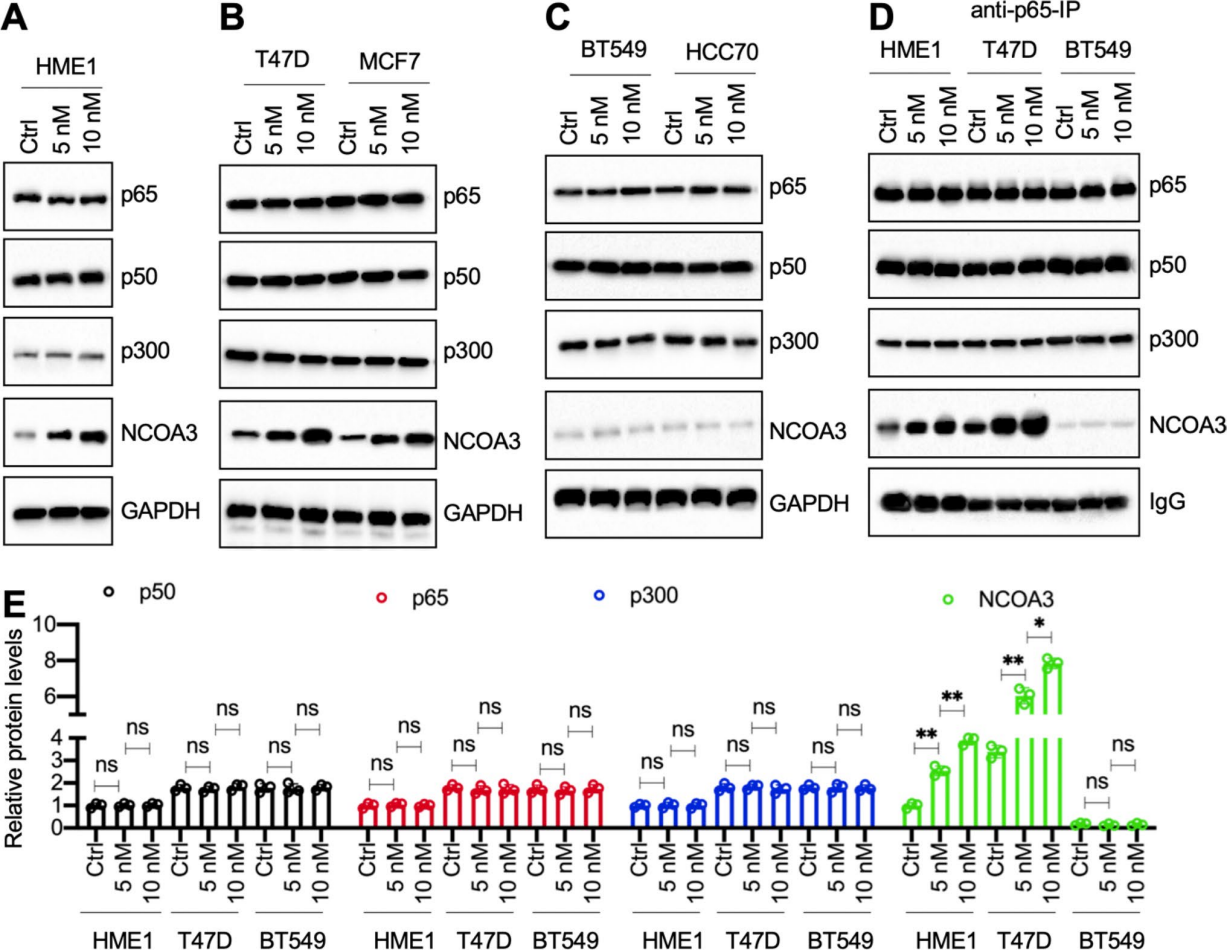
E2 dose-dependently induced the expression of NCOA3. Four cell lines (HME1, T47D, BT549, and HCC70) were treated with different concentrations (0, 5, and 10 nM in PBS) of E2 for 4 h. Th MCF7 cell line was co-treated with 20 nM MLN4924 and different concentrations (0, 5, and 10 nM in PBS) of E2 for 4 h. Cells were used for protein isolation and immunoblots to examine the protein levels of p65, p50, p300, NCOA3, and GAPDH (loading control). **A** Immunoblot results in HME1 cells. **B** Immunoblot results in T47D and MCF7 cells. **C** Immunoblot results in BT549 and HCC70 cells. **D** Immunoprecipitation results. The HME1, T47D, and BT549 cells were treated with different concentrations of E2 (0, 5, and 10 nM in PBS) for 4 h, followed by immunoprecipitation using anti-p65-coupled protein A agarose. The purified p65-interacting proteins were probed to detect the protein levels of p65, p50, p300, and NCOA3. IgG was used as a loading control. For all immunoblot experiments in this figure, three independent replicates were performed. For each lane in a replicate, three independent protein samples were mixed with equal weights (20 μg for each). One group of immunoblot images is shown. **E** Quantified protein levels. The protein levels of p50, p65, p300, and NCOA3 in (**D**) were normalized to their corresponding IgG. The quantified results in (**E**) represent the means of three replicates ± SD. Significant differences were determined by one-way ANOVA, followed by Tukey's post hoc test. ns: no significant difference; * *P* < 0.05 and ** *P* < 0.01

We next investigated whether E2 treatment affected the assembly of the NCOA3-p300-NF-κB complex. We first treated HME1, T47D, MCF7, BT549, and HCC70 cells with 5 nM or 10 nM E2. The immunoblot results showed that E2 treatments dose-dependently induced the protein expression of NCOA3 but did not change the protein levels of p65, p50, or p300 in HME1 cells and in two ER-positive cell lines (Fig. 4A, B, S13A, and S13B). The E2 treatments did not change the protein levels of any of the NCOA3-p300-NF-κB complex members in the two ER-negative cell lines (Fig. 4C and S13C). Immunoprecipitation assays using anti-p65-coupled protein A agarose in E2-treated HME1, T47D, and BT549 cells also showed that E2 treatment could dose-dependently increase the amounts of NCOA3 protein immunoprecipitated by p65 in the HME1 and T47D cells, but not in the BT549 cells (Fig. 4D and E).

### Overexpression of NCOA3-p300-NF-κB members upregulated the expression of *BCL2*, *BCL2A1*, *BCL2L2*, and *MCL1*

We also verified that the NCOA3-p300-NF-κB complex mediated the expression of *BCL2*, *BCL2A1*, *BCL2L2*, and *MCL1,* but not of *BCL2L1*, by overexpressing p65, p50, p300, and NCOA3 in T47D cells (Figure S14A–F). This overexpression of the NCOA3-p300-NF-κB complex members resulted in a significant upregulation of the expression of *BCL2*, *BCL2A1*, *BCL2L2*, and *MCL1,* but not of *BCL2L1* (Figure S14G).

We also examined the mRNA levels of *NCOA3* in human ER-negative and ER-positive tumor samples. As expected, the average expression level of *NCOA3* was significantly higher in ER-positive tumors than in ER-negative tumors (Figure S15A). Pearson’s correlation analysis of the gene expression levels indicated that *NCOA3* expression was positively correlated with the expression of *ERα*, *ERβ, BCL2*, *BCL2A1*, *BCL2L2*, and *MCL1,* but not with *BCL2L1* expression (Figure S15B–H).

### The NCOA3-p300-NF-κB complex mediated the expression of *BCL2*, *BCL2A1*, *BCL2L2*, and *MCL1* by binding to their promoters

**Fig. 5:**
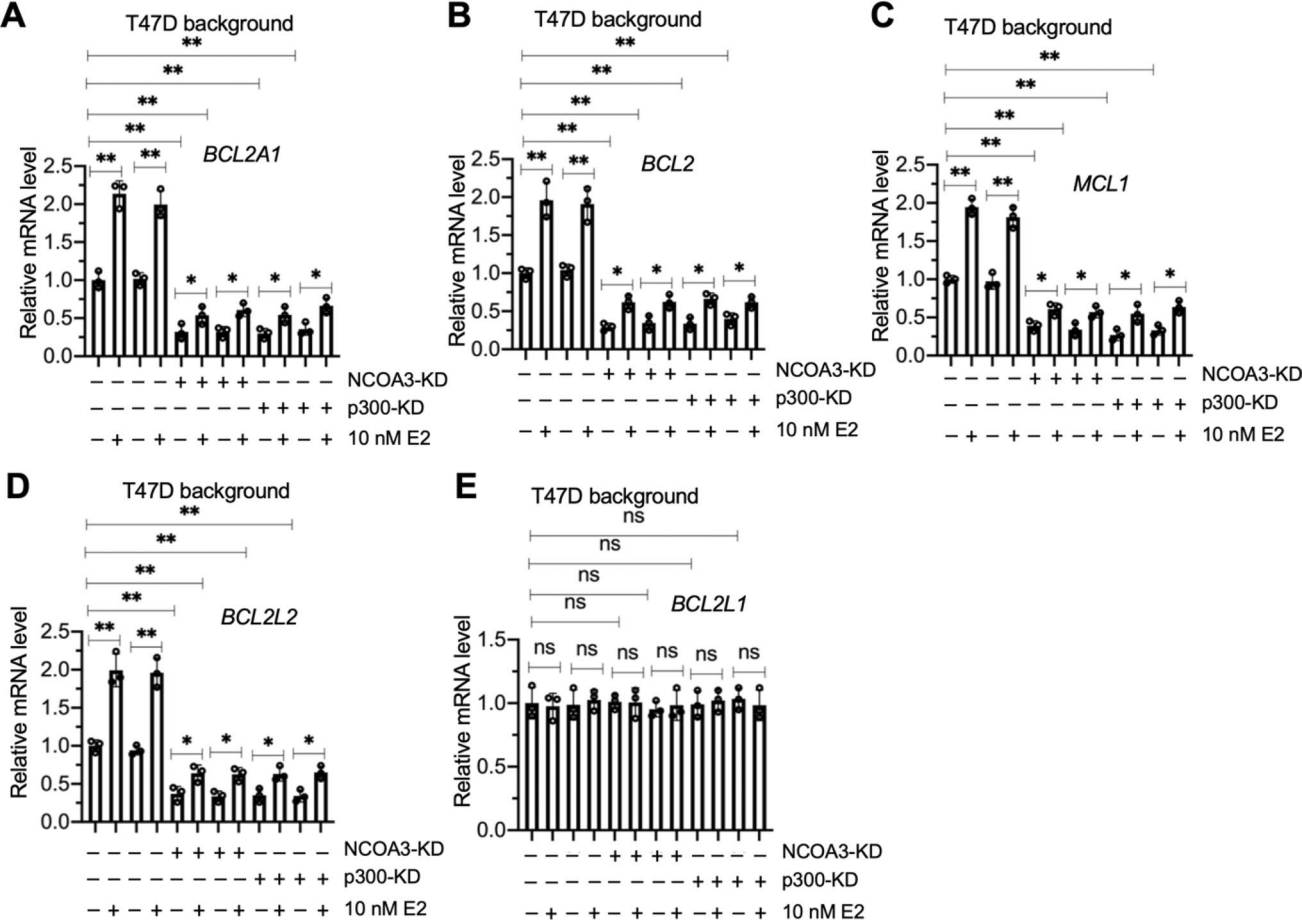
Knockdown of NCOA3 and p300 in T47D cells resulted in the suppression of *BCL2A1*, *BCL2*, *MCL1*, and *BCL2L2*. The Control-KD1/2, NCOA3-KD1/2, and p300-KD1/2 cells (T47D background) were treated with 10 nM E2 and without E2 (used PBS) for 4 h, followed by RNA isolation and RT-qPCR analyses to measure the mRNA levels of *BCL2A1* (**A**), *BCL2* (**B**), *MCL1* (**C**), *BCL2L2* (**D**), and *BCL2L1* (**E**). Three independent replicates (n = 3 for each replicate) were performed, and the results represent the means of three replicates ± SD. Significant differences were determined by one-way ANOVA, followed by Tukey's post hoc test. ns: no significant difference; * *P* < 0.05 and ** *P* < 0.01

Since NF-κB was required for the regulation of *BCL2*, *BCL2A1*, *BCL2L2*, and *MCL1*, we next determined whether p300 and NCOA3 had similar regulatory effects on these antiapoptotic genes. We generated two independent cell lines for p300 and NCOA3 knockdown in the T47D background (Figure S16). As observed following knockdown of NF-κB subunits, depletion of p300 and NCOA3 also downregulated *BCL2*, *BCL2A1*, *BCL2L2*, and *MCL1,* but not *BCL2L1* (Fig. 5A–E). E2 treatment slightly reversed the suppression of *BCL2*, *BCL2A1*, *BCL2L2*, and *MCL1* (Fig. 5A–E).

We then performed ChIP assays with the Control-KD1, RelA-KD1, NFKB1-KD1, p300-KD1, and NCOA3-KD1 cells (T47D background) with or without 10 nM E2 treatment to determine the changes in the occupancy of the NCOA3-p300-NF-κB complex on the *BCL2*, *BCL2A1*, *BCL2L2*, and *MCL1* promoters. The NCOA3-p300-NF-κB complex bound to the promoters of *BCL2*, *BCL2A1*, *BCL2L2*, and *MCL1,* but not *BCL2L1* (Figures S17 and S18)*.* Depletion of any member of the NCOA3-p300-NF-κB complex decreased the occupancies of the other two members on the promoters of *BCL2*, *BCL2A1*, *BCL2L2*, and *MCL1* (Figures S17 and S18). In RelA-KD1, NFKB1-KD1, p300-KD1, and NCOA3-KD1 cells, E2 treatment slightly increased the occupancies of NCOA3-p300-NF-κB members on the promoters of *BCL2*, *BCL2A1*, *BCL2L2*, and *MCL1* compared to the occupancies observed in untreated KD cells (Figures S17 and S18).

### Depletion of the NCOA3-p300-NF-κB members significantly inhibited breast cancer cell growth in vitro and in vivo

**Fig. 6:**
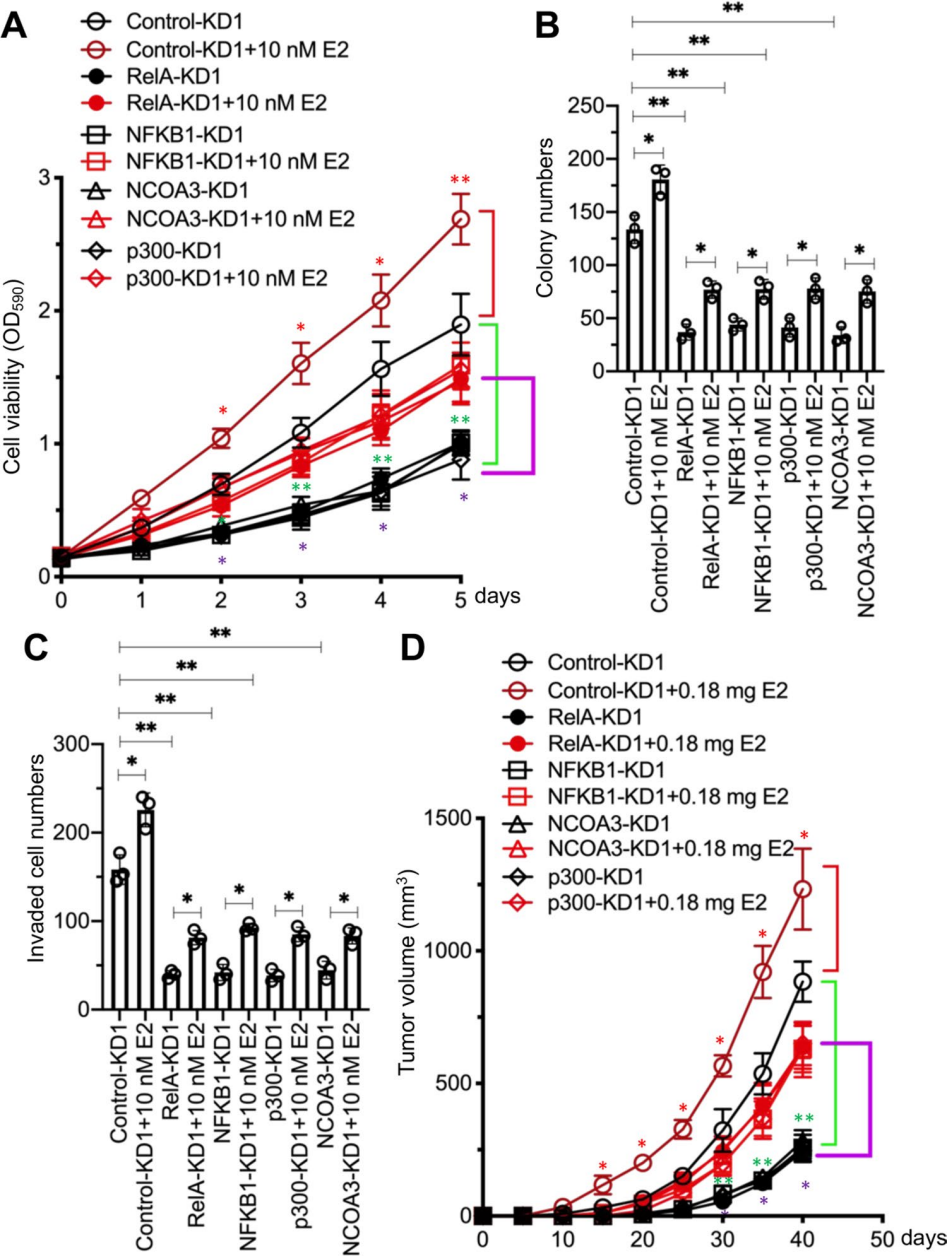
Deficiency of NCOA3-p300-NF-κB members inhibited breast cancer cell proliferation, invasion, and in vivo tumor growth. **A** Cell viability. The Control-KD1, p65-KD1, p50-KD1, p300-KD1, and NCOA3-KD1 cells were used for the MTT assay after treatment with PBS or 10 nM E2 in PBS at different time points (0, 1, 2, 3, 4, and 5 days). Three independent replicates (n = 3 for each replicate) were performed, and the results represent the means of three replicates ± SD. **B** Colony numbers. Cells in (**A**) were used for the colony formation assay. **C** Invaded cell numbers. Cells in (**A**) were used for the cell invasion assay. For experiments in (**B**) and (**C**), three independent replicates (n = 3 for each replicate) were performed, and the results represent the means of three replicates ± SD. **D** Tumor volumes. Cells in (**A**) were injected into female nude mice (n = 10 for each cell line), followed by implantation or no implantation of a 0.18 mg E2 pellet. Tumor volumes were measured at 5-day intervals for 40 days. Three independent replicates (n = 10 for each replicate) were performed, and the results represent means of three replicates ± SD. Significant differences in this figure were determined by one-way ANOVA, followed by Tukey's post hoc test. * *P* < 0.05 and ** *P* < 0.01

Since depletion of NCOA3-p300-NF-κB members in breast cancer cells caused the downregulation of antiapoptotic genes, we next investigated whether depletion of NCOA3-p300-NF-κB members would have an anti-proliferative/anti-survival effect on breast cancer cells. Using Control-KD1, RelA-KD1, NFKB1-KD1, p300-KD1, and NCOA3-KD1 cells in a T47D background, and treating with or without 10 nM E2, we performed both short-term cell proliferation and long-term clonogenic assays. As expected, we observed that depletion of NCOA3-p300-NF-κB members inhibited cell proliferation and colony formation (Figs. 6A, B, and S19A). E2 treatment slightly reversed this inhibition of cell proliferation and colony formation (Fig. 6A and B). Knockdown of NCOA3-p300-NF-κB components dramatically inhibited cell invasion, and E2 treatment partially restored this ability (Figs. 6C and S19B).

We determined the protein levels of p65, p50, p300, NCOA3, Bcl2-A1, Bcl2, Mcl1, Bcl2-w, Bcl2-xL, and three apoptotic markers (Bak, Bax, and caspase-9) in these cells. Consistent with their mRNA levels, the protein levels of Bcl2-A1, Bcl2, Mcl1, and Bcl2-w were significantly decreased in cells with knockdown of NCOA3-p300-NF-κB components (Figure S20). Conversely, the protein levels of Bak, Bax, and cleaved caspase-9 were significantly increased following the depletion of NCOA3-p300-NF-κB components (Figure S20). E2 treatment partially increased the antiapoptotic protein levels but decreased the apoptotic protein levels (Figure S20). These findings provided the first demonstration of an effect of E2 on the expression of the components of the NCOA3-p300-NF-κB complex and its downstream apoptotic molecules.

We also evaluated the antitumor effects of knockdown of NCOA3-p300-NF-κB components by establishing a tumor xenograft model by injecting Control-KD1, RelA-KD1, NFKB1-KD1, p300-KD1, or NCOA3-KD1 cells (T47D background) into nude mice. Tumor growth was significantly slower in mice bearing RelA-KD1, NFKB1-KD1, p300-KD1, and NCOA3-KD1 cells than in mice injected with Control-KD1 cells (Fig. 6D). Implantation of a 0.18 mg E2 pellet into mice injected with NCOA3-p300-NF-κB component-KD cells partially restored the inhibited tumor growth (Fig. 6D).

### Two NCOA3 inhibitors blocked NCOA3 function and decreased the expression of antiapoptotic genes

Several NCOA3 inhibitors, such as gossypol and bufalin (Figure S21A), are commercially available and show strong abilities to disrupt the function of NCOA3 [24]. We treated T47D cells with different concentrations of gossypol (0, 1, 2.5, 5, and 10 μM) and bufalin (0, 25, 50, 100, and 200 nM) to determine the effects of these inhibitors on NCOA3. We found that 5 μM gossypol and 100 nM bufalin caused a nearly 65–70% decrease in NCOA3 levels (Figures S21B–E), and we used these two concentrations in further evaluations of the function of NCOA3.

**Fig. 7:**
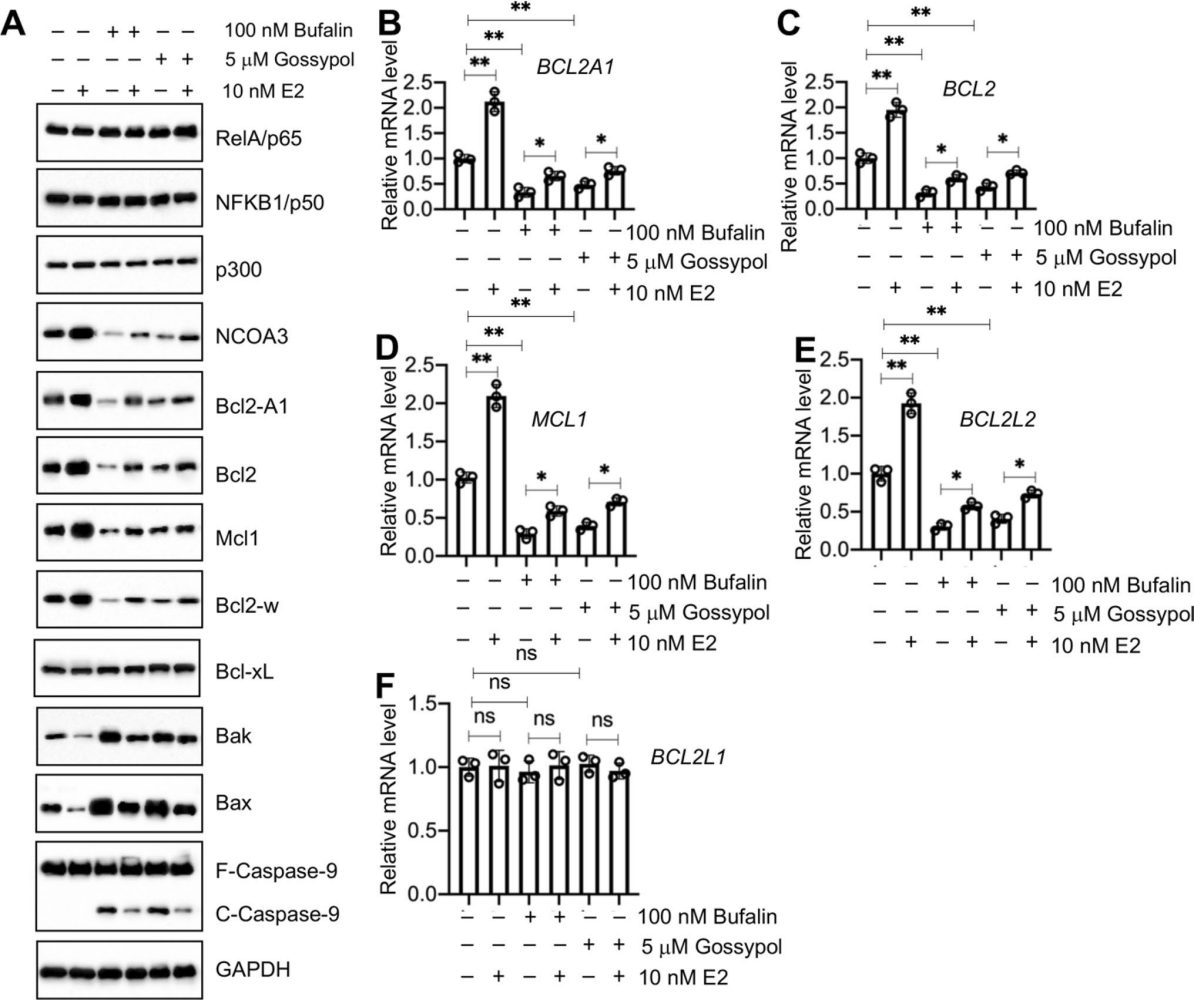
Treatments with NCOA3 inhibitors caused the dysfunction of NCOA3 and the suppression of its target antiapoptotic genes. **A** The protein levels of NCOA3-p300-NF-κB members and antiapoptotic and apoptotic proteins in T47D cells treated with NCOA3 inhibitors. T47D cells were treated with ( +) or without (−, treated with PBS) 100 nM bufalin and 5 μM gossypol for 6 h, followed by additional treatment with ( +) or without (−, treated with PBS) 10 nM E2 for 4 h. Cells were then lysed and used for immunoblots to detect the protein levels of p65, p50, NCOA3, p300, Bcl2A1, Bcl2, Mcl1, Bcl-w, Bcl-xL Bak, Bax, caspase-9, and GAPDH (loading control). Three independent replicates were performed. For each lane in a replicate, three independent protein samples were mixed with equal weights (20 μg each). One representative group of immunoblot images is shown. **B**–**F** The mRNA levels of antiapoptotic genes. Total RNA samples from cells in (**A**) were used for RT-qPCR analyses to measure the mRNA levels of *BCL2A1* (**B**), *BCL2* (**C**), *MCL1* (**D**), *BCL2L2* (**E**), and *BCL2L1* (**F**). Three independent replicates (n = 3 for each replicate) were performed, and the results represent the means of three replicates ± SD. Significant differences were determined by one-way ANOVA, followed by Tukey's post hoc test. ns: no significant difference; ** *P* < 0.01 and *** *P* < 0.001

We examined whether NCOA3 inhibitors would inhibit antiapoptotic gene expression by treating T47D cells with 100 nM bufalin or 5 μM gossypol, followed by additional treatment with 10 nM E2. The immunoblot results showed that treatments with either inhibitor caused a decrease in NCOA3 protein levels but had no effect on the protein levels of p65, p50, or p300 (Fig. 7A). Similar to the results in NCOA3-KD cells, we also observed decreases in Bcl2-A1, Bcl2, Mcl1, and Bcl2-w, but increases in Bax, Bak, and cleaved caspase-9 in the cells treated with either gossypol or bufalin (Fig. 7A). E2 treatment slightly reversed the inhibition of antiapoptotic protein expression (Fig. 7A). Similar to the protein levels, we also observed suppression of expression of *BCL2*, *BCL2A1*, *BCL2L2*, and *MCL1,* but not *BCL2L1,* in T47D cells treated with either inhibitor (Fig. 7B–F). Treatment of bufalin- or gossypol-treated cells with E2 only slightly induced the expression of *BCL2*, *BCL2A1*, *BCL2L2*, and *MCL1* (Fig. 7B–E).

ChIP assays performed on T47D cells co-treated with NCOA3 inhibitors and 10 nM E2 using anti-NCOA3- and IgG-coupled coupled protein G agarose showed that either bufalin or gossypol markedly decreased the occupancy of NCOA3 on the promoters of *BCL2*, *BCL2A1*, *BCL2L2*, and *MCL1* (Figure S22). Treatment with 10 nM E2 only slightly increased the occupancy of NCOA3 (Figure S22).

### NCOA3 inhibitors significantly inhibited breast cancer cell growth in vitro and in vivo

We next investigated whether breast cancer cells treated with NCOA3 inhibitors would exhibit any anti-proliferative/anti-survival effects. We treated T47D cells with 100 nM bufalin or 5 μM gossypol, and then determined in vitro cell proliferation, colony formation, and cell invasion. As expected, we observed that both NCOA3 inhibitors significantly inhibited the cell proliferation, colony formation, and cell invasion of T47D cells (Figures S23A–C and S24). E2 treatment of NCOA3 inhibitor-treated cells slightly reversed the inhibition of cell proliferation, colony formation, and cell invasion (Figures S23A–C and S24).

We also evaluated the antitumor effects of NCOA3 inhibitors by establishing a tumor xenograft model by injecting T47D cells and then comparing mice with or without implanted 0.18 mg E2 pellets. After tumor volumes reached 150 mm^3^, the mice with or without E2 implants were randomly divided into three subgroups: Control group (injection with PBS), bufalin group (injection with 1.5 mg/kg bufalin), and gossypol group (injection with 50 mg/kg gossypol), and we monitored the progression of tumor proliferation at 5 day intervals. Tumor growth was significantly slower in mice administered bufalin or gossypol than in control mice injected with PBS in both the E2-implanted and non-implanted conditions (Figure S23D).

## Discussion

**Fig. 8:**
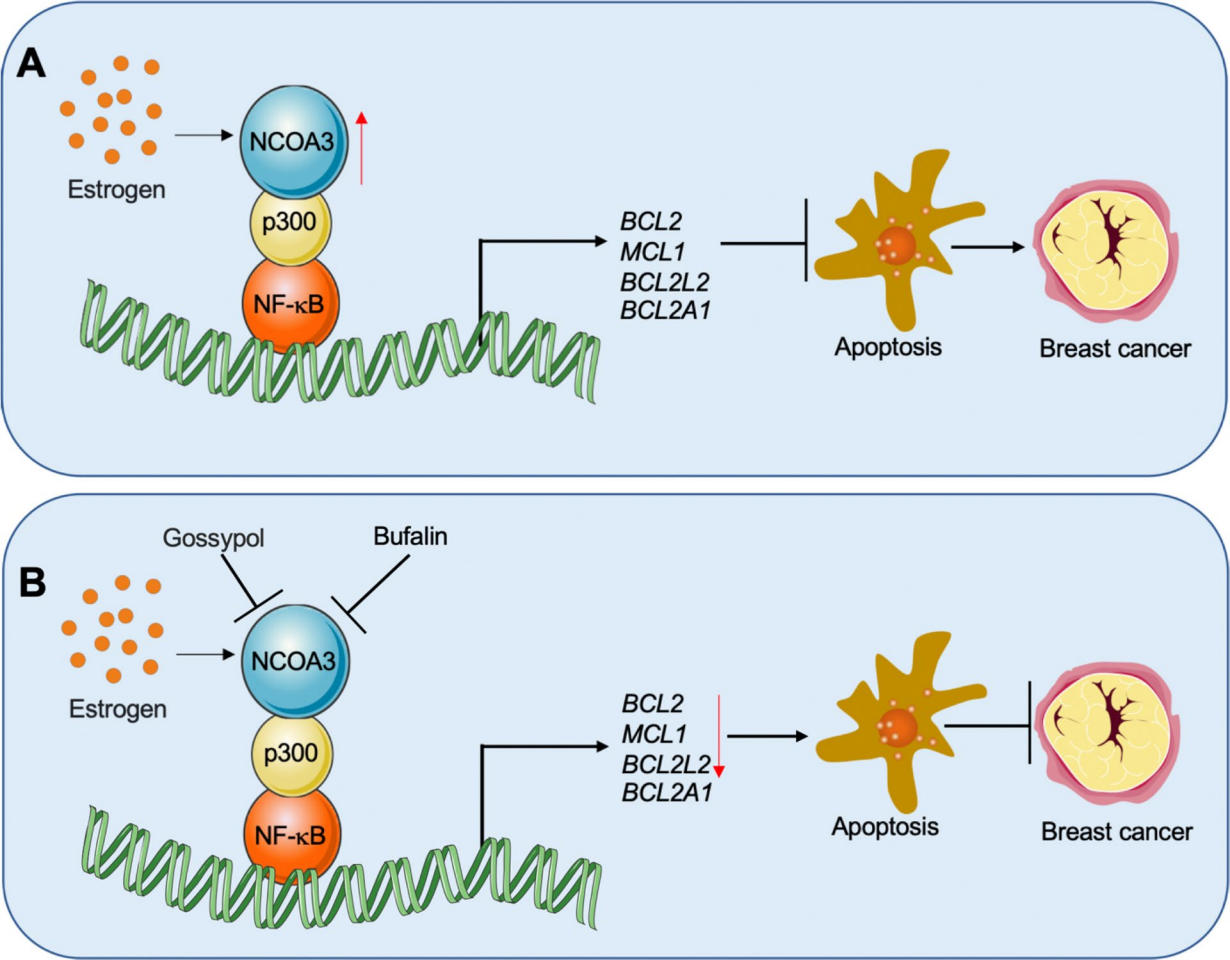
Schematic diagrams of the NCOA3-p300-NF-κB complex in the regulation of antiapoptotic genes during breast cancer pathogenesis. **A** Schematic diagram of the role of the NCOA3-p300-NF-κB complex in the regulation of antiapoptotic genes. NF-κB binds to the promoters of *BCL2A1*, *BCL2*, *MCL1*, and *BCL2L2,* where it recruits p300 and estrogen-activated NCOA3 to form a complex. NCOA3 functions as a coactivator to transactivate the expression of *BCL2A1*, *BCL2*, *MCL1*, and *BCL2L2*. The induced antiapoptotic proteins inhibit the apoptosis process and cause uncontrolled cell proliferation, triggering tumorigenesis. **B** Targeting the NCOA3-p300-NF-κB complex with NCOA3 inhibitors to suppress the expression of antiapoptotic genes. Bufalin and gossypol impair the function of NCOA3, thereby blocking the function of the NCOA3-p300-NF-κB complex and causing the suppression of *BCL2A1*, *BCL2*, *MCL1*, and *BCL2L2* expression and eventually inhibiting breast cancer cell growth

Although upregulation of antiapoptotic genes has been observed for decades in breast cancer cells [5, 8], the underlying mechanisms of how these genes are activated are still under investigation. The results presented here have led us to propose a schematic model that explains how NCOA3 coordinates with p300 and NF-κB to transactivate antiapoptotic genes in breast cancer cells. We revealed that NF-κB recruits p300, and that p300 then binds to NCOA3 to form a transcriptional complex capable of docking on the promoters of *BCL2*, *BCL2A1*, *BCL2L2*, and *MCL1* to increase the expression of these genes. Upregulation of antiapoptotic genes inhibits apoptosis and results in uncontrolled cell proliferation, thereby triggering breast cell tumorigenesis (Fig. 8A). NCOA3-specific inhibitors, such as bufalin and gossypol, block NCOA3 function, thereby negatively affecting the function of the NCOA3-p300-NF-κB complex and consequently decreasing the expression of *BCL2*, *BCL2A1*, *BCL2L2*, and *MCL1*. Downregulation of these antiapoptotic genes then promotes apoptosis and inhibits breast cancer cell growth (Fig. 8B).

The proapoptotic or antiapoptotic Bcl-2 family members are required for the maintenance of cellular function [25]. The Bax/Bak homodimers and heterodimers permeabilize the outer mitochondrial membrane upon the activation of Bid, Bim, and Puma, leading to the leakage of cytochrome *c* and activation of caspase cascades [25, 26]. Antiapoptotic proteins interact with Bax/Bak, inhibiting the oligomerization or activation of Bax/Bak and preventing the release of cytochrome *c* from the mitochondria [25, 26]. Dysregulations of apoptotic and antiapoptotic proteins are important triggers of breast tumorigenesis [25, 26].

At present, the molecular mechanisms by which these genes are activated remain unclear. Several published papers have found that NF-κB binds to the promoters of *BCL2*, *BCL2A1*, and *MCL1* and activates their expression in many cell types, such as prostate cancer cells [15], esophageal squamous carcinoma cells [16], and fibrosarcoma cells [17]. Currently, two main questions remain unanswered regarding the overexpression of antiapoptotic genes in breast cancer cells. The first question is whether antiapoptotic genes are overexpressed in all types of breast cancer cells or only in specific cell types. The second is how these antiapoptotic genes are regulated at the transcriptional level. In this study, we revealed that *BCL2*, *BCL2A1*, *BCL2L2*, and *MCL1,* but not *BCL2L1,* are significantly overexpressed in ER-positive breast cancer cells, whereas their expression is only slightly increased in ER-negative cancer cells. We also revealed that estrogen-activated NCOA3 functions as a transcriptional coactivator that forms a complex with p300 and two NF-κB subunits, and that this complex binds to the promoters of *BCL2*, *BCL2A1*, *BCL2L2*, and *MCL1* to transactivate their expression. To our knowledge, our study is the first to resolve the question of how antiapoptotic genes are regulated by the NCOA3-p300-NF-κB complex. Our results provide new insight into the regulation of antiapoptotic gene expression in different cells.

Gene expression is controlled by transcriptional complexes assembled by transcription factors and regulators [27]. NCOA3 overexpression has been observed in breast cancer cells, and mice that overexpress NCOA3 show increased cell proliferation and can generate breast tumors [19]. Here, we identified NCOA3 as a transcriptional coactivator that responds to estrogen, and NCOA3 has been identified as a coactivator of placenta-specific 1 (*PLAC1*) in ER-positive breast cancer cells [19]. However, the NCOA3-p300-NF-κB complex functions as a mediator of the expression of four antiapoptotic genes in HME1 cells (with low ER levels) and in ER-positive breast cancer cells, suggesting that this regulatory mechanism is not cancer-specific. We speculate two different mechanisms may explain this phenomenon. First, the NCOA3-p300-NF-κB complex-mediated regulation may exist in all ER-positive cells (either normal cells or cancer cells). The expression levels of *BCL2*, *BCL2A1*, *BCL2L2*, and *MCL1* in HME1 were similar to those in HMEC2.6 cells, which may reflect inefficient induction of NCOA3 by low ER levels, and that would limit the function of NCOA3-p300-NF-κB. Second, the ER may be involved in the regulation of *BCL2*, *BCL2A1*, *BCL2L2*, and *MCL1* expression.

Among the 5 antiapoptotic genes, only *BCL2L1* was not regulated by the NCOA3-p300-NF-κB complex. The overexpression of *BCL2L1* in both ER-positive and ER-negative breast cancer cell lines suggested that *BCL2L1* was not dependent on estrogen. Depletion of any member of the NCOA3-p300-NF-κB complex did not change the expression of *BCL2L1,* suggesting that *BCL2L1* might be controlled by a different transcriptional complex or complexes.

Some publications have shown that E2 treatment caused the downregulation of ERα in MCF7 cells [28, 29]. However, the underlying mechanism of ERα downregulation is still being investigated. We also observed E2-dependent downregulation of ERα in MCF7 cells at the beginning of this study. We investigated the underlying mechanism and found that E2 treatment simultaneously upregulated ERα and activated an E3 ligase called SCF^FBXL12^. This E3 ligase ubiquitinated and degraded ERα. MLN4924, an inhibitor of SCF E3 ligases, blocked the degradation of ERα (unpublished data).

Pharmacological inhibition of Bcl2 family proteins was initially considered an effective strategy in cancer therapy [7], and several Bcl2 protein inhibitors, such as ABT-199, ABT-263, ABT-737, and AT-101, have been developed as cancer therapeutics [7]. However, clinical and preclinical studies have revealed that pharmacological inhibition of Bcl-2 and Bcl-xL alone in breast cancer cells causes the induction of Mcl1, which then leads to drug resistance and failure to inhibit tumor growth [5–7]. In the current study, depletion of NCOA3-p300-NF-κB members and NCOA3 inhibitors simultaneously decreased the expression levels of *BCL2*, *BCL2A1*, *BCL2L2*, and *MCL1*, suggesting that targeting NCOA3 or the assembly of the NCOA3-p300-NF-κB complex might be a new and effective approach for inhibiting breast tumor growth. The present standard of care for ER-positive breast cancer is hormone therapy using drugs like tamoxifen and toremifene [30]. Exploration of the combined use of NCOA3 inhibitors and endocrine therapy could be very meaningful in the treatment of ER-positive patients or in the investigation of the therapeutic effects of NCOA3 inhibitors in endocrine therapy-resistant patients in the future.

In summary, we demonstrated that estrogen-activated NCOA3 couples with p300 and NF-κB to transactivate antiapoptotic genes, thereby inhibiting apoptosis in ER-positive breast cancer cells. Inhibition of the NCOA3 function or specific knockdown of NCOA3 expression significantly attenuated the expression of antiapoptotic genes, promoted apoptosis, and inhibited ER-positive breast cancer cell growth. Our studies identified NCOA3 inhibitors as novel agents capable of inducing apoptosis in ER-positive breast cancer cells. Taken together, our findings have revealed a new mechanism that explains how antiapoptotic genes are activated during tumorigenesis.

## Supplementary Information

Supplementary file1 (PDF 16117 KB)

Supplementary file2 (DOCX 27 KB)

## Data Availability

The datasets during and/or analyzed during the current study available from online public databases or the corresponding authors on reasonable request.
