# Supplementary material for: Estrogen-dependent activation of NCOA3 couples with p300 and NF-κB to mediate antiapoptotic genes in ER-positive breast cancer cells
Source: Discov Oncol. 2023 Feb 28;14:28. doi: 10.1007/s12672-023-00635-0 (PMC9975134; doi:10.1007/s12672-023-00635-0)
Supplement: Supplementary file 1 — Supplementary file1 (PDF 16117 KB) [file 12672_2023_635_MOESM1_ESM.pdf]

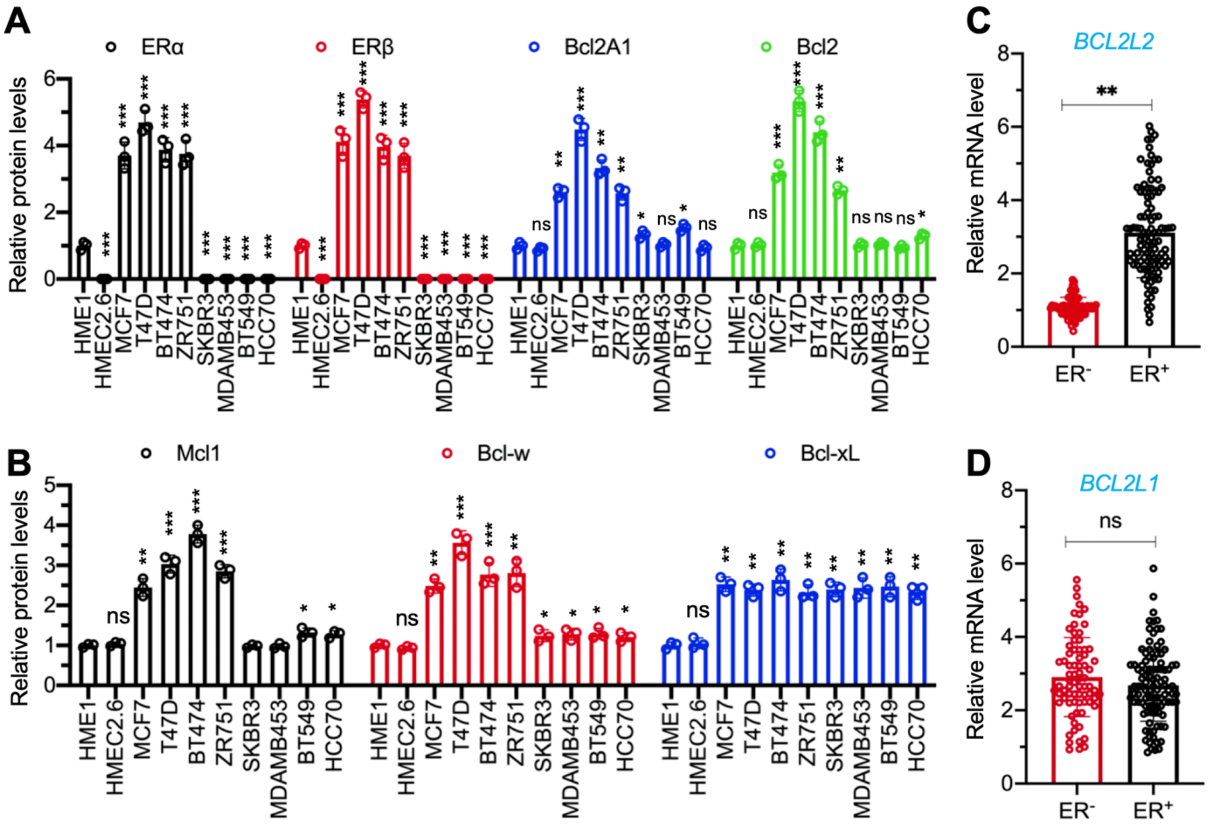

Figure S1. The quantified protein levels of ER and antiapoptotic proteins in breast cancer cell lines and the mRNA levels of *BCL2L1/2* in clinical ER-negative and ER-positive tumors

(A and **B)** The quantified protein levels of ERα, ERβ, and antiapoptotic proteins in breast cancer cell lines**.** Protein signals in Figure 1C were quantified using Image J software and then normalized to their corresponding loading control (GAPDH). Protein levels in HME1 were defined as one-fold and protein levels in other cells were all compared with those in HME1. **(A)** ERα, ERβ, Bcl2A1, and Bcl2. **(B)** Mcl1, Bcl-w, and Bcl-xL. Results represented means of three replicates ± SD. Significant difference was determined by the One-way ANOVA, followed by Tukey's post-hoc test. ns: no significant difference; * $P < 0.05$, ** $P < 0.01$, and *** $P < 0.001$. **(C** and **D)** The mRNA levels of *BCL2L1/2* in ER-negative and

ER-positive tumor samples. The same RNA samples as used in Figure 1D were subjected to detect mRNA levels of *BCL2L2* **(C)** and *BCL2L1* **(D)**. Three independent replicates were performed and one group of representative results was shown. Significant difference was determined by the One-way ANOVA, followed by Tukey's post-hoc test. ns: no significant difference; ** $P < 0.01$.

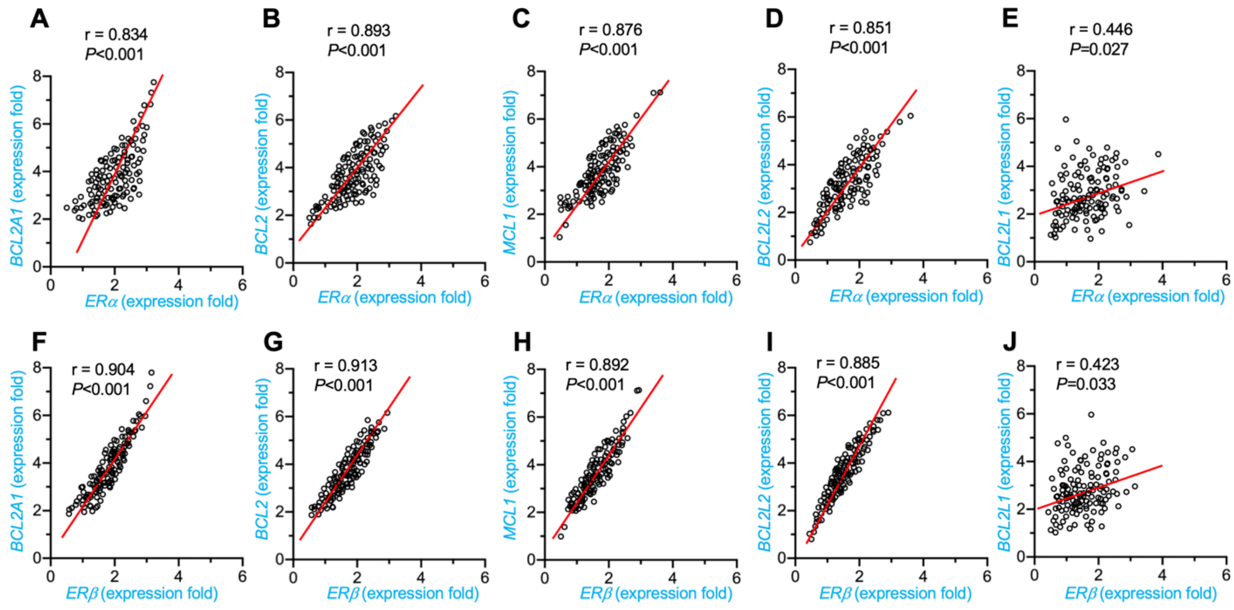

Figure S2. Expression levels of four antiapoptotic gene were positively correlated with *ERα/ERβ*

The expression levels of antiapoptotic genes (*BCL2A1*, *BCL2*. *MCL1*, *BCL2L2*, and *BCL2L1*) and *ERα/ERβ* in ER-positive tumor samples (n=102) were used for Pearson's correlation with SPSS software. **(A)** Correlation between *BCL2A1* and *ERα.* **(B)** Correlation between *BCL2* and *ERα.* **(C)** Correlation between *MCL1* and *ERα.* **(D)** Correlation between *BCL2L2* and *ERα.* **(E)** Correlation between *BCL2L1* and *ERα.* **(F)** Correlation between *BCL2A1* and *ERβ.* **(G)** Correlation between *BCL2* and *ERβ.* **(H)** Correlation between *MCL1* and *ERβ.* **(I)** Correlation between *BCL2L2* and *ERβ.* **(J)** Correlation between *BCL2L1* and *ERβ.* Three independent replicates were performed and one group of representative results was shown.

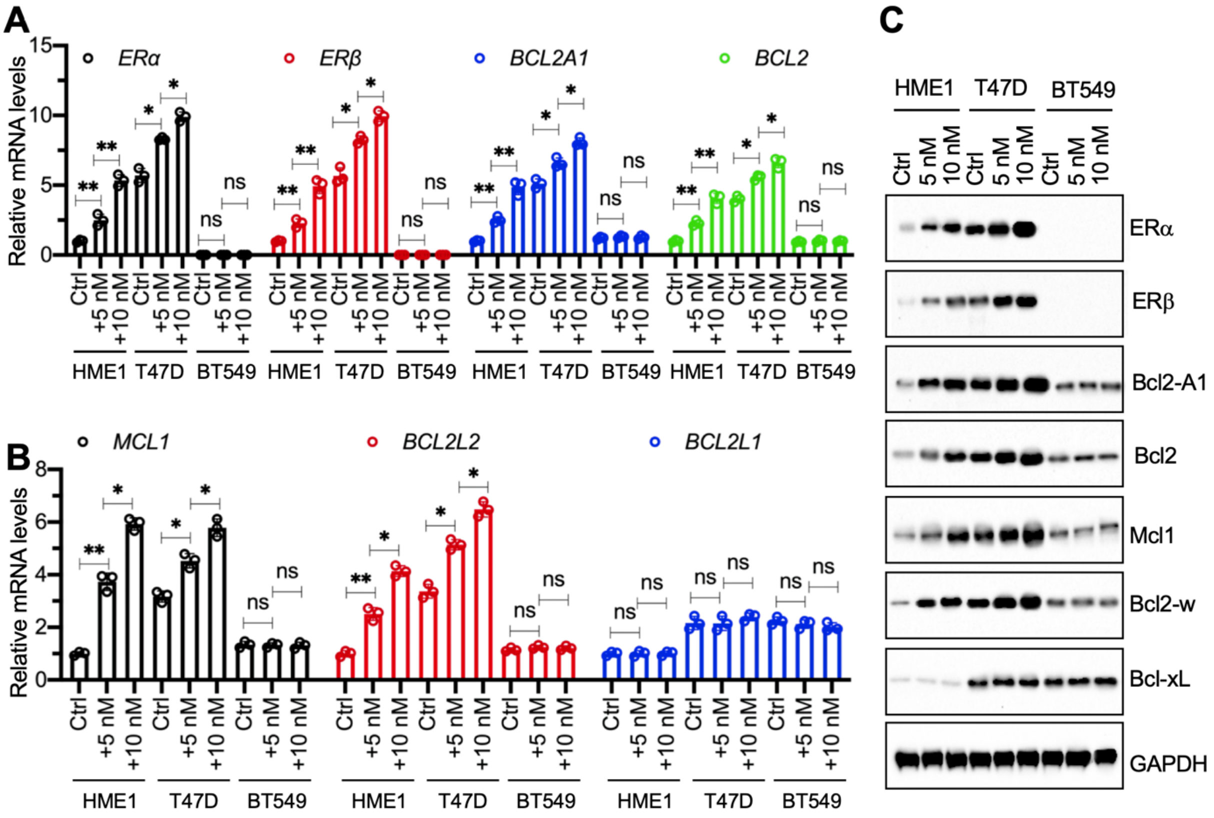

Figure S3. E2 treatments dose-dependently induced the mRNA and protein levels of *BCL2A1*, *BCL2*. *MCL1* and *BCL2L2* in HME1, T47D and BT549 cells

(A and **B)** The mRNA levels of *ERα*, *ERβ*, and antiapoptotic genes in cells treated with or without E2**.** The HME1, T47D, and BT549 cells were treated with PBS (Ctrl), 5 nM, or 10 nM E2 for 4 h. Total RNA samples from these cells were used for RT-qPCR analyses to measure mRNA levels of genes. **(A)** *ERα*, *ERβ*, *BCL2A1*, and *BCL2*. **(B)** *MCL1*, *BCL2L2*, and *BCL2L1*. The expression levels of these 7 genes in PBS-treated HME1 cells were defined as one-fold and their expression levels in other cell lines were all compared to those in PBS-treated HME1 cells. Three independent replicates (n=3 for each replicate) were performed and results represented means of three replicates ± SD. Significant difference was determined by the One-way ANOVA, followed by Tukey's post-hoc test.

ns: no significant difference; * $P < 0.05$ and ** $P < 0.01$. **(C)** The protein levels of ER and antiapoptotic proteins. Total protein extracts from cells in (A) were used for immunoblots to measure the protein levels of ERα, ERβ, Bcl2A1, Bcl2, Mcl1, Bcl-w, Bcl-xL, and GAPDH (loading control). Three independent replicates were performed. For each lane in a replicate, three independent protein samples were mixed with equal weights (20 μg for each). One group of immunoblot images were shown.

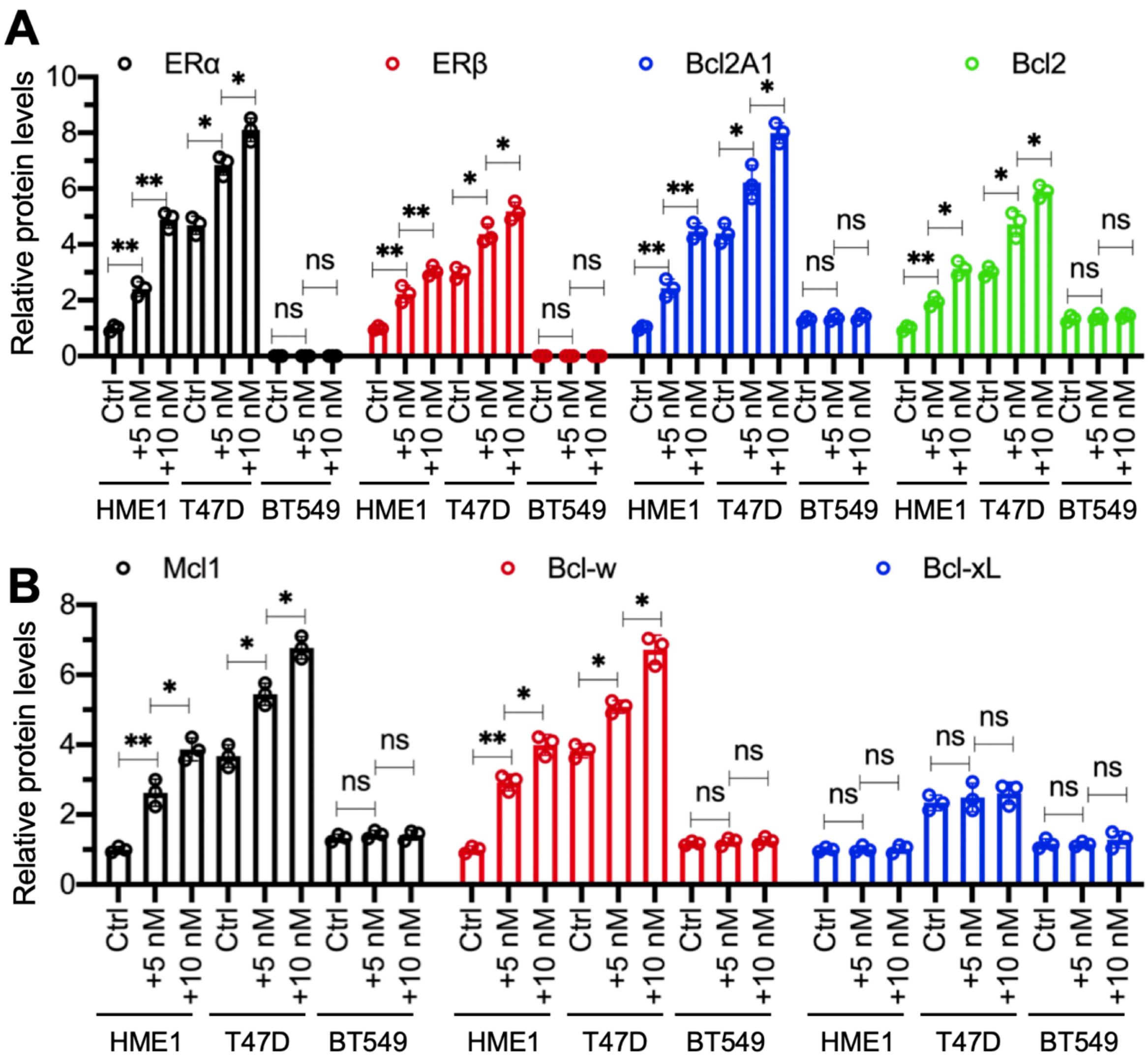

Figure S4. The quantified protein levels of ER and antiapoptotic proteins in HME1, T47D and BT549 cells treated with or without E2

Protein signals in Figure S3C were quantified using Image J software and then normalized to their corresponding loading control (GAPDH). Protein levels in PBS-treated HME1 (Ctrl) were defined as one-fold and protein levels in other cells were all compared with those in PBS-treated HME1. **(A)** ERα, ERβ, Bcl2A1, and Bcl2. **(B)** Mcl1, Bcl-w, and Bcl-xL. Results represented means of three replicates ± SD. Significant difference was

determined by the One-way ANOVA, followed by Tukey's post-hoc test. ns: no significant difference; * $P < 0.05$ and ** $P < 0.01$.

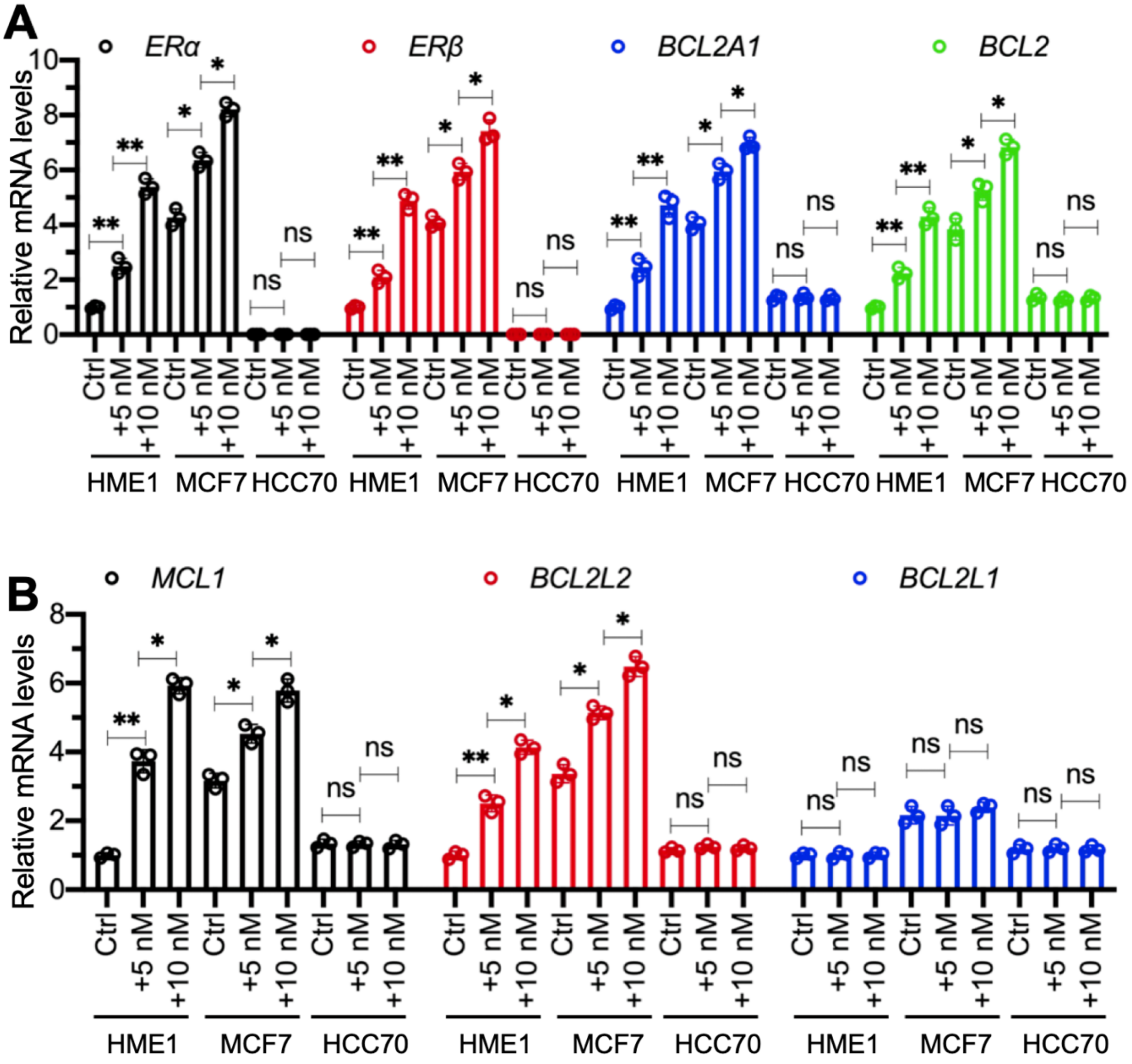

Figure S5. E2 treatments dose-dependently induced the mRNA levels of *BCL2A1*, *BCL2*. *MCL1* and *BCL2L2* in HME1, MCF7 and HCC70 cells

(A and **B)** The mRNA levels of *ERα*, *ERβ*, and antiapoptotic genes in cells treated with or without E2**.** The HME1, MCF7 and HCC70 cells were treated with PBS (Ctrl), 5 nM, or 10 nM E2 for 4 h. Total RNA samples from these cells were used for RT-qPCR analyses to measure mRNA levels of genes. **(A)** *ERα*, *ERβ*, *BCL2A1*, and *BCL2*. **(B)** *MCL1*, *BCL2L2*, and *BCL2L1*. The expression levels of these 7 genes in PBS-treated HME1 cells

were defined as one-fold and their expression levels in other cell lines were all compared to those in PBS-treated HME1 cells. Three independent replicates (n=3 for each replicate) were performed and results represented means of three replicates ± SD. Significant difference was determined by the One-way ANOVA, followed by Tukey's post-hoc test. ns: no significant difference; * $P < 0.05$ and ** $P < 0.01$.

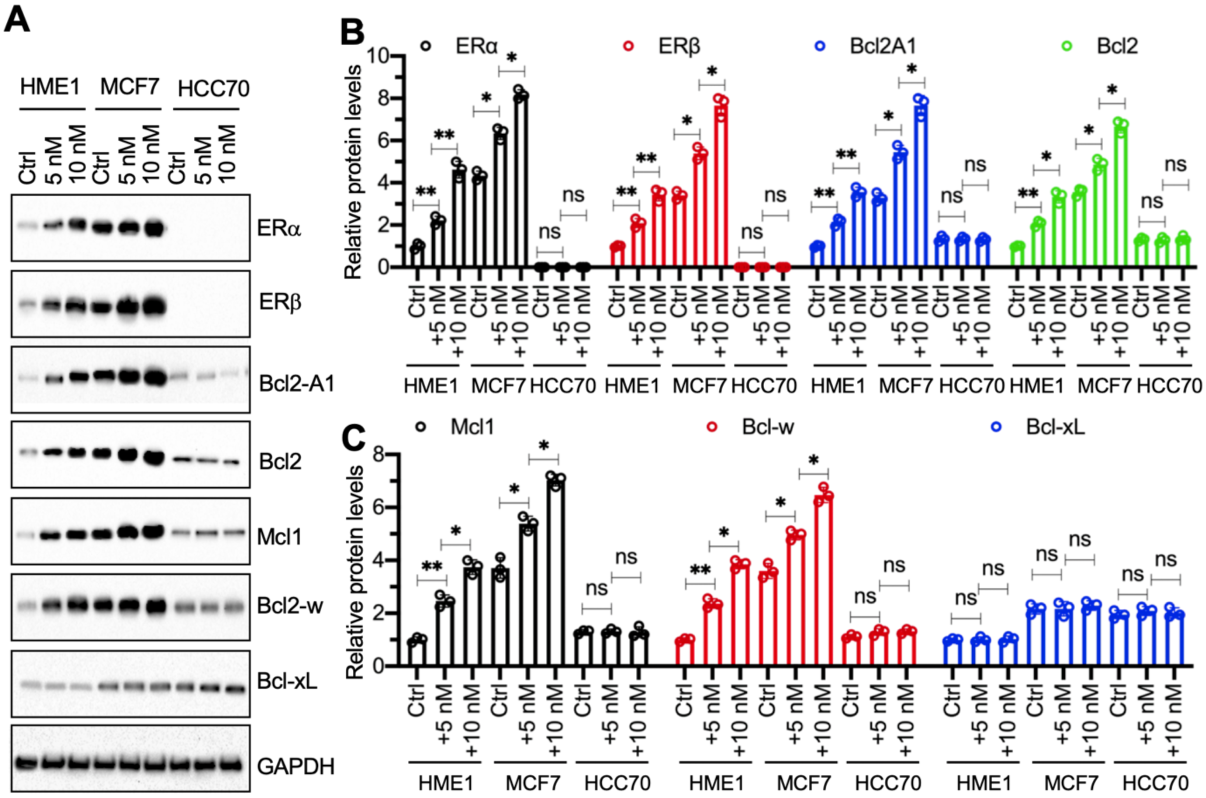

Figure S6. E2 treatments dose-dependently induced the protein levels of Bcl2A1, and Bcl2. Mcl1, Bcl-w in HME1, MCF7 and HCC70 cells

(A) The protein levels of ER and antiapoptotic proteins. Total protein extracts from cells in Figure S5 were used for immunoblots to measure the protein levels of ERα, ERβ, Bcl2A1, Bcl2, Mcl1, Bcl-w, Bcl-xL, and GAPDH (loading control). Three independent replicates were performed. For each lane in a replicate, three independent protein samples were mixed with equal weights (20 μg for each). One group of immunoblot images were shown. **(B and C)** Quantified protein levels. Protein signals in (A) were quantified using Image J software and then normalized to their corresponding loading control (GAPDH). Protein levels in PBS-treated HME1 (Ctrl) were defined as one-fold and protein levels in other cells were all compared with those in PBS-treated HME1. **(B)** ERα, ERβ, Bcl2A1, and Bcl2. **(C)** Mcl1, Bcl-w, and Bcl-xL. Results represented means of three

replicates ± SD. Significant difference was determined by the One-way ANOVA, followed by Tukey's post-hoc test. ns: no significant difference; * $P < 0.05$ and ** $P < 0.01$.

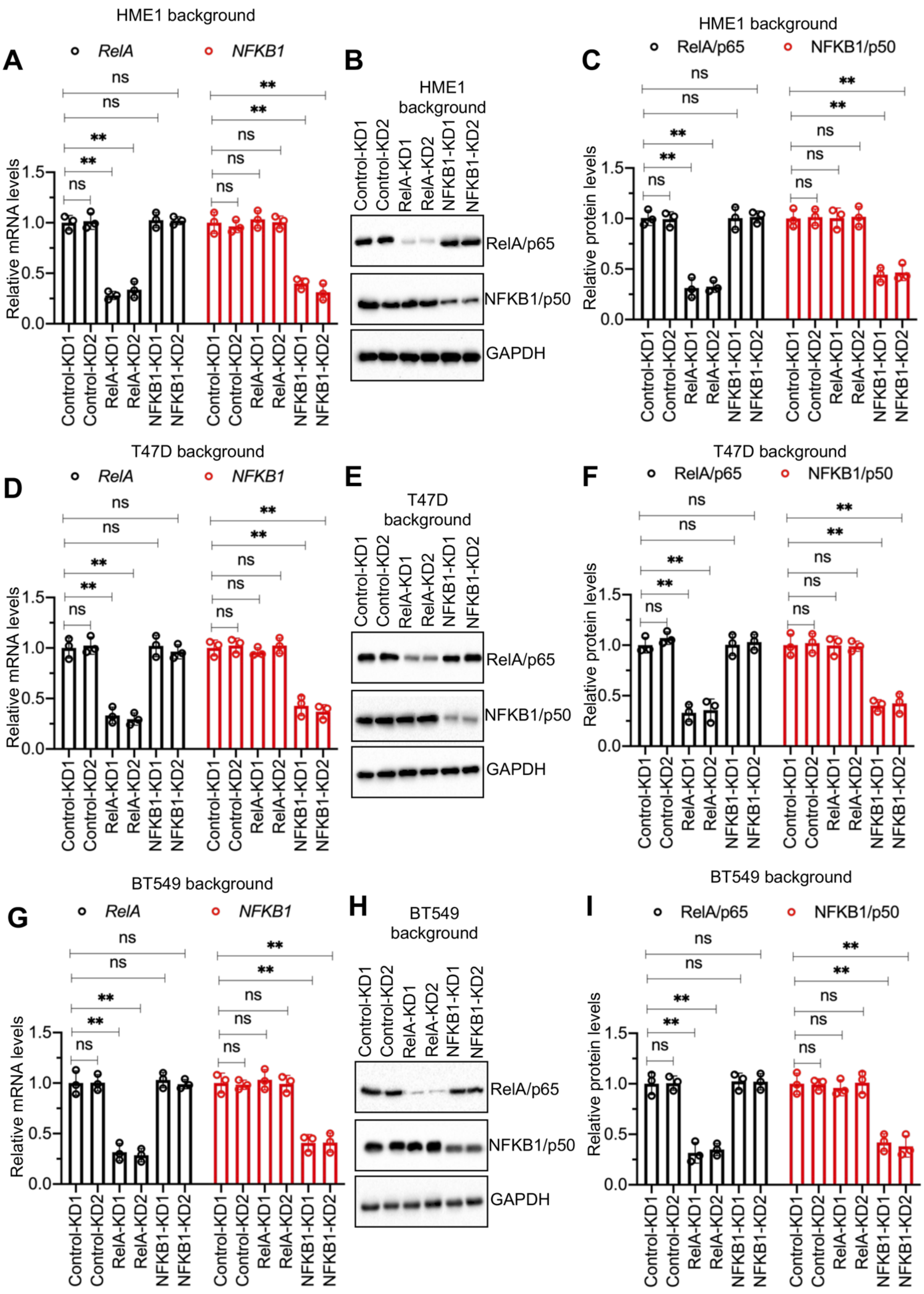
HME1 background
A
RelA
NFKB1
ns
**
Relative mRNA levels
1.5
1.0
0.5
0.0
Control-KD1
Control-KD2
RelA-KD1
RelA-KD2
NFKB1-KD1
NFKB1-KD2
B
HME1 background
RelA/p65
NFKB1/p50
GAPDH
C
HME1 background
RelA/p65
NFKB1/p50
Relative protein levels
T47D background
D
E
T47D background
F
T47D background
BT549 background
G
H
BT549 background
I
BT549 background

Figure S7. The mRNA and protein levels of RelA and NFKB1 in RelA-KD and NFKB1-KD cells in HME1, T47D and BT549 backgrounds

(A) The mRNA levels of *RelA* and *NFKB1* in Control-KD1/2, RelA-KD1/2 and NFKB1-KD1/2 cells (HME1 background). **(B** and **C)** The protein levels of p65 and p50 in Control-KD1/2, RelA-KD1/2 and NFKB1-KD1/2 cells (HME1 background). **(B)** Western blotting results. **(C)** Quantified protein levels. **(D)** The mRNA levels of *RelA* and *NFKB1* in Control-KD1/2, RelA-KD1/2 and NFKB1-KD1/2 cells (T47D background). **(E** and **F)** The protein levels of p65 and p50 in Control-KD1/2, RelA-KD1/2 and NFKB1-KD1/2 cells (T47D background). **(E)** Western blotting results. **(F)** Quantified protein levels. **(G)** The mRNA levels of *RelA* and *NFKB1* in Control-KD1/2, RelA-KD1/2 and NFKB1-KD1/2 cells (BT549 background). **(H** and **I)** The protein levels of p65 and p50 in Control-KD1/2, RelA-KD1/2 and NFKB1-KD1/2 cells (BT549 background). **(H)** Western blotting results. **(I)** Quantified protein levels. For the measurement of mRNA levels in this figure, the expression levels of *RelA* and *NFKB1* genes in Control-KD1 cells in each background were defined as one-fold and their expression levels in other cell lines were all compared to those in Control-KD cells (each background). Three independent replicates (n=3 for each replicate) were performed and results represented means of three replicates ± SD. Significant difference was determined by the One-way ANOVA, followed by Tukey's post-hoc test. ns: no significant difference; ** $P < 0.01$. For immunoblots, three independent replicates were performed. For each lane in a replicate, three independent protein samples were mixed with equal weights (20 μg for each). One group of immunoblot images were shown. For quantification of protein levels, protein signals were quantified using Image J software and then normalized to their corresponding loading control (GAPDH). Protein levels in

Control-KD1 cells in each background were defined as one-fold and protein levels in other cells were all compared with those in Control-KD1 (each background). Results represented means of three replicates ± SD. Significant difference was determined by the One-way ANOVA, followed by Tukey's post-hoc test. ns: no significant difference; ** $P < 0.01$.

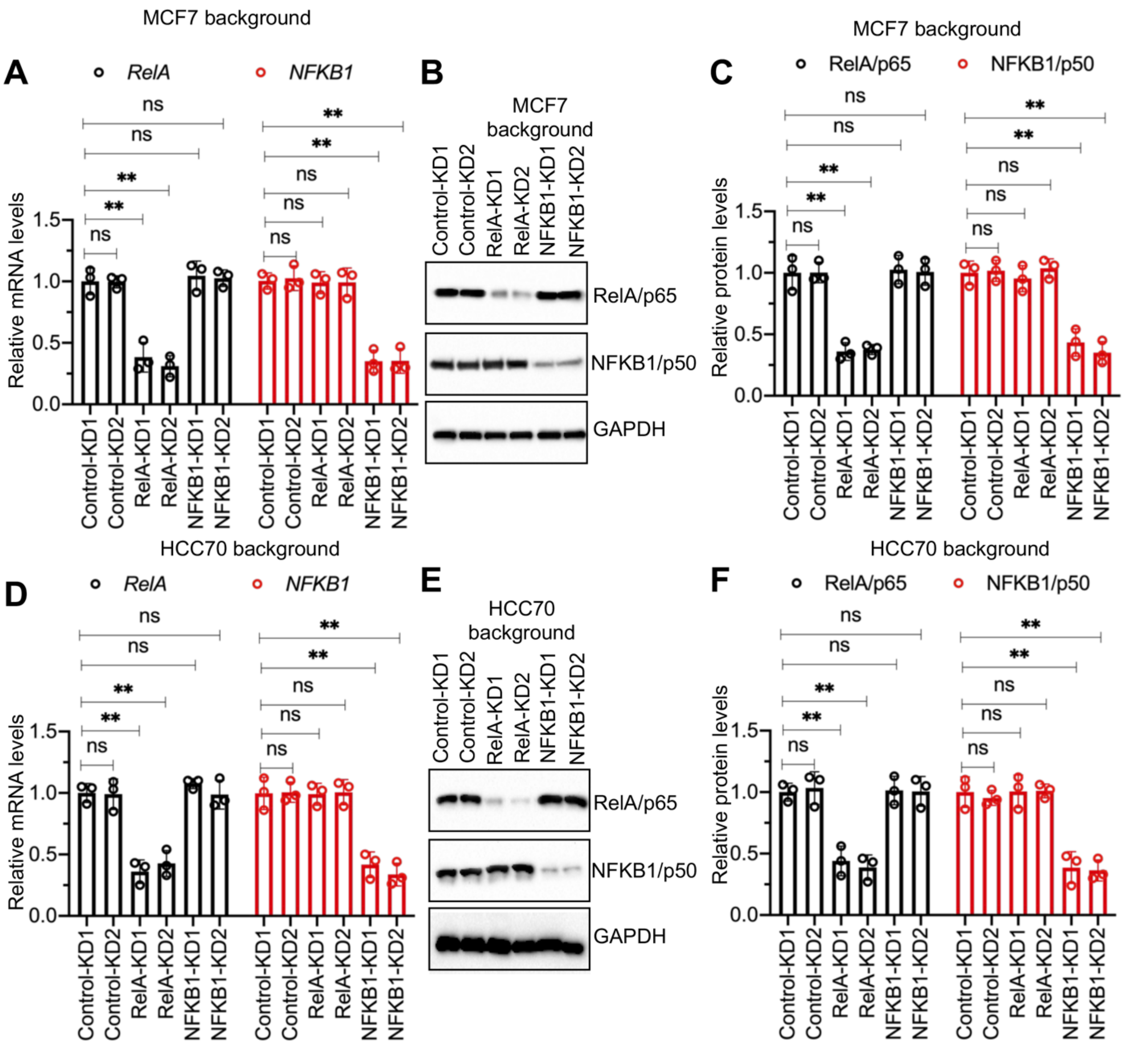

Figure S8. The mRNA and protein levels of RelA and NFKB1 in RelA-KD and NFKB1-KD cells in MCF7 and HCC70 backgrounds

(A) The mRNA levels of *RelA* and *NFKB1* in Control-KD1/2, RelA-KD1/2 and NFKB1-KD1/2 cells (MCF7 background). **(B** and **C)** The protein levels of p65 and p50 in Control-KD1/2, RelA-KD1/2 and NFKB1-KD1/2 cells (MCF7 background). **(B)** Western blotting results. **(C)** Quantified protein levels. **(D)** The mRNA levels of *RelA* and *NFKB1* in Control-KD1/2, RelA-KD1/2 and NFKB1-KD1/2 cells (HCC70 background). **(E** and **F)** The protein levels of p65 and p50 in Control-KD1/2, RelA-KD1/2 and NFKB1-KD1/2 cells (HCC70

background). **(E)** Western blotting results. **(F)** Quantified protein levels. For the measurement of mRNA levels in this figure, the expression levels of *RelA* and *NFKB1* genes in Control-KD1 cells in each background were defined as one-fold and their expression levels in other cell lines were all compared to those in Control-KD cells (each background). Three independent replicates (n=3 for each replicate) were performed and results represented means of three replicates ± SD. Significant difference was determined by the One-way ANOVA, followed by Tukey's post-hoc test. ns: no significant difference; ** $P < 0.01$. For immunoblots, three independent replicates were performed. For each lane in a replicate, three independent protein samples were mixed with equal weights (20 μg for each). One group of immunoblot images were shown. For quantification of protein levels, protein signals were quantified using Image J software and then normalized to their corresponding loading control (GAPDH). Protein levels in Control-KD1 cells in each background were defined as one-fold and protein levels in other cells were all compared with those in Control-KD1 (each background). Results represented means of three replicates ± SD. Significant difference was determined by the One-way ANOVA, followed by Tukey's post-hoc test. ns: no significant difference; ** $P < 0.01$.

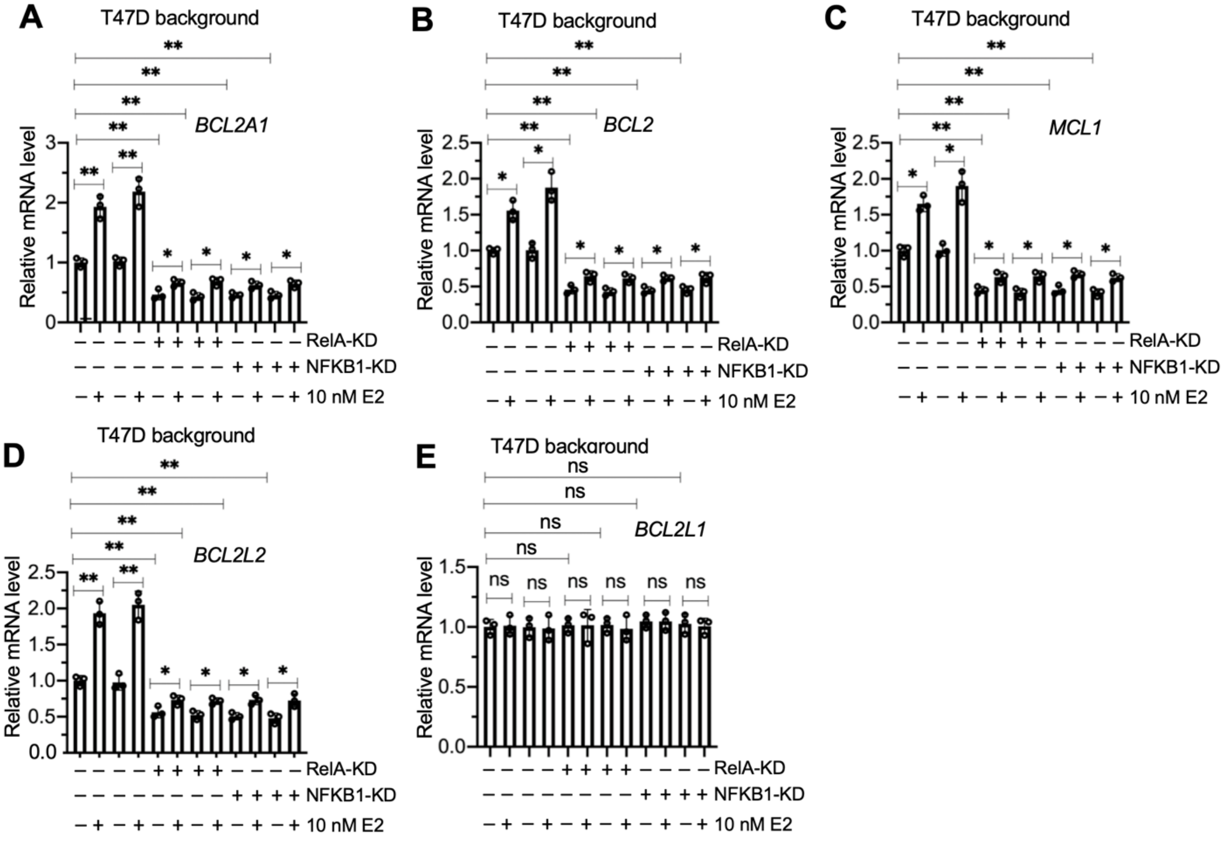

Figure S9. The expression levels of *BCL2A1*, *BCL2*, *MCL1*, and *BCL2L2* in RelA-KD and NFKB1-KD cells (T47D background) treated with or without E2

The Control-KD1/2, RelA-KD1/2, and NFKB1-KD1/2 cells in T47D background were treated with 10 nM E2 or without E2 (used PBS) for 4 h, followed by RNA isolation and RT-qPCR analyses to measure mRNA levels of *BCL2A1* **(A)**, *BCL2* **(B)**, *MCL1* **(C)**, *BCL2L2* **(D)**, and *BCL2L1* **(E)**. The expression levels of these 5 genes in PBS-treated Control-KD1 cells were defined as one-fold and their expression levels in other cell lines/conditions were all compared to those in the PBS-treated Control-KD1 cells. Three independent replicates (n=3 for each replicate) were performed and results represented means of three replicates ± SD. Significant difference was determined by the One-way

ANOVA, followed by Tukey's post-hoc test. ns: no significant difference; * $P < 0.05$ and ** $P < 0.01$.

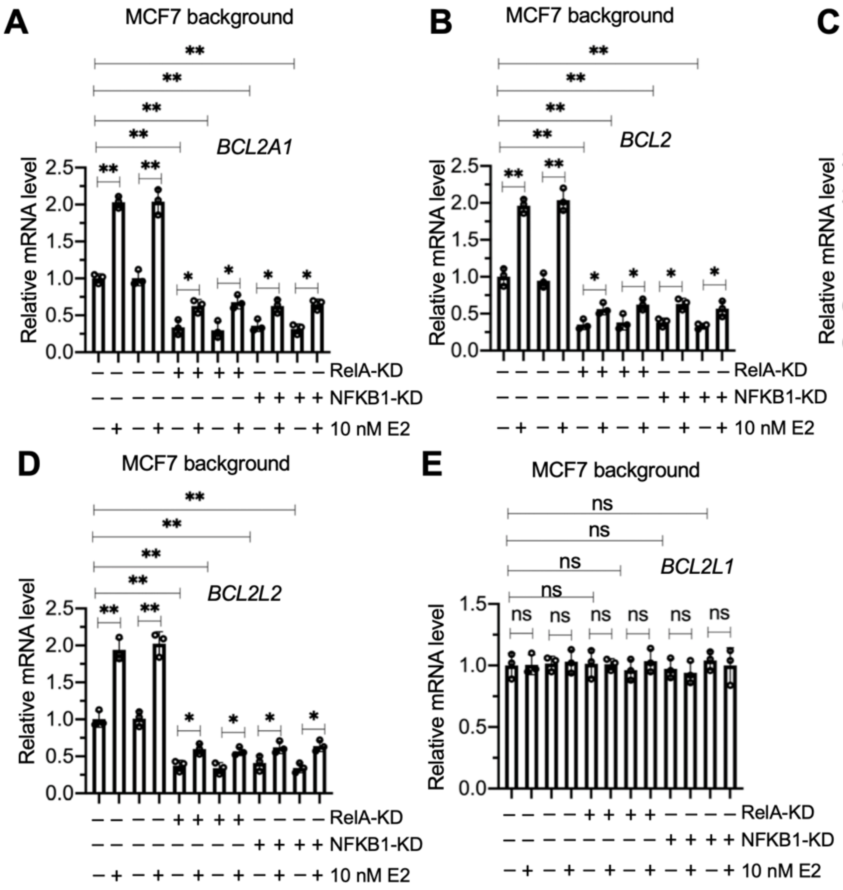

Figure S10. The expression levels of *BCL2A1*, *BCL2*, *MCL1*, and *BCL2L2* in RelA-KD and NFKB1-KD cells (MCF7 background) treated with or without E2

The Control-KD1/2, RelA-KD1/2, and NFKB1-KD1/2 cells in MCF7 background were treated with 10 nM E2 or without E2 (used PBS) for 4 h, followed by RNA isolation and RT-qPCR analyses to measure mRNA levels of *BCL2A1* **(A)**, *BCL2* **(B)**, *MCL1* **(C)**, *BCL2L2* **(D)**, and *BCL2L1* **(E)**. The expression levels of these 5 genes in PBS-treated Control-KD1 cells were defined as one-fold and their expression levels in other cell lines/conditions were all compared to those in the PBS-treated Control-KD1 cells. Three independent replicates (n=3 for each replicate) were performed and results represented means of three replicates ± SD. Significant difference was determined by the One-way

ANOVA, followed by Tukey's post-hoc test. ns: no significant difference; * $P < 0.05$ and ** $P < 0.01$.

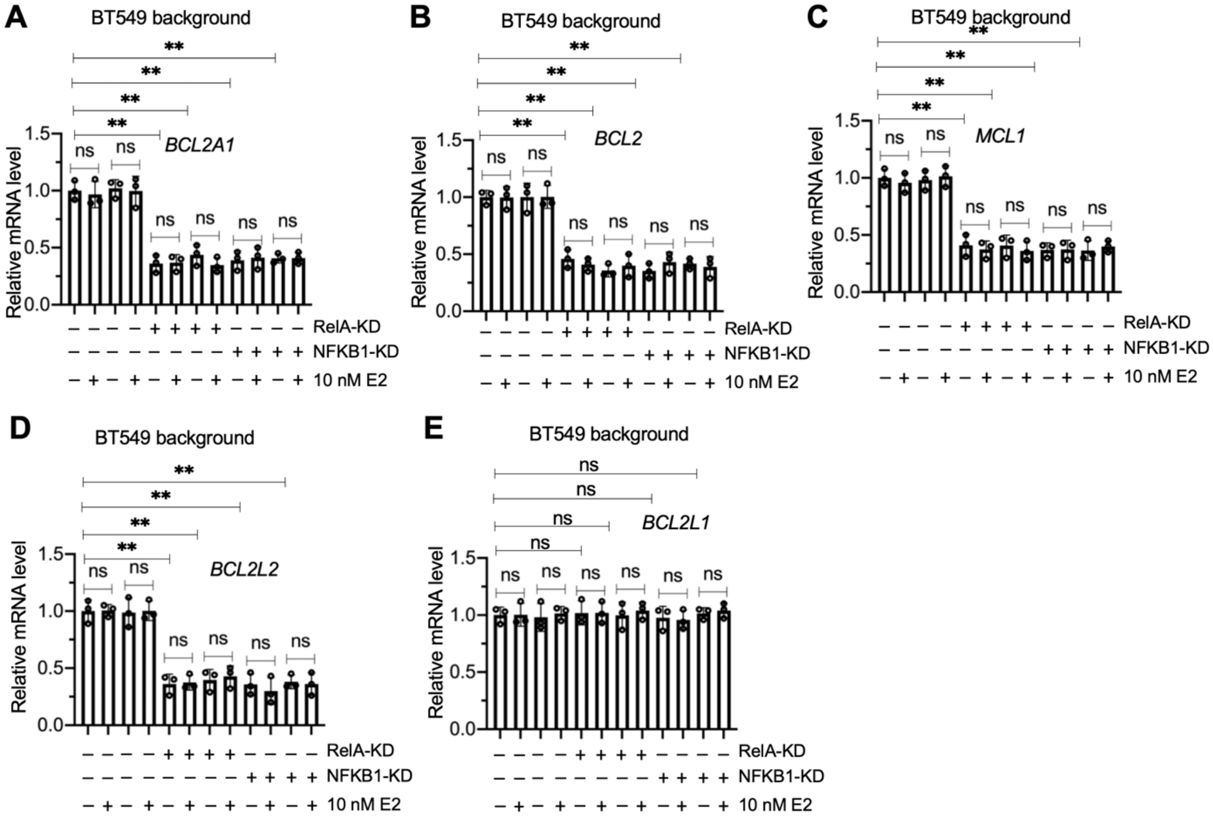

Figure S11. The expression levels of *BCL2A1*, *BCL2*, *MCL1*, and *BCL2L2* in RelA-KD and NFKB1-KD cells (BT549 background) treated with or without E2

The Control-KD1/2, RelA-KD1/2, and NFKB1-KD1/2 cells in BT549 background were treated with 10 nM E2 or without E2 (used PBS) for 4 h, followed by RNA isolation and RT-qPCR analyses to measure mRNA levels of *BCL2A1* **(A)**, *BCL2* **(B)**, *MCL1* **(C)**, *BCL2L2* **(D)**, and *BCL2L1* **(E)**. The expression levels of these 5 genes in PBS-treated Control-KD1 cells were defined as one-fold and their expression levels in other cell lines/conditions were all compared to those in the PBS-treated Control-KD1 cells. Three independent replicates (n=3 for each replicate) were performed and results represented means of three replicates ± SD. Significant difference was determined by the One-way ANOVA, followed by Tukey's post-hoc test. ns: no significant difference.

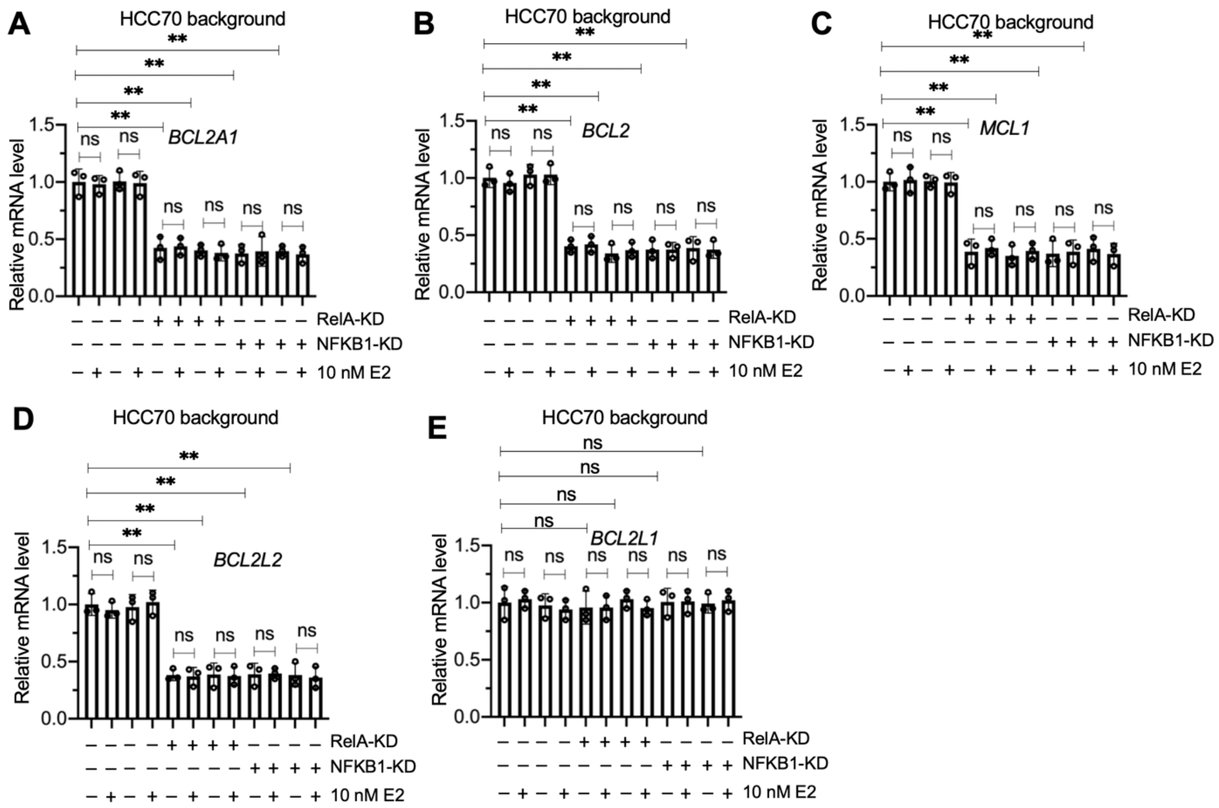

Figure S12. The expression levels of *BCL2A1*, *BCL2*, *MCL1*, and *BCL2L2* in RelA-KD and NFKB1-KD cells (HCC70 background) treated with or without E2

The Control-KD1/2, RelA-KD1/2, and NFKB1-KD1/2 cells in HCC70 background were treated with 10 nM E2 or without E2 (used PBS) for 4 h, followed by RNA isolation and RT-qPCR analyses to measure mRNA levels of *BCL2A1* **(A)**, *BCL2* **(B)**, *MCL1* **(C)**, *BCL2L2* **(D)**, and *BCL2L1* **(E)**. The expression levels of these 5 genes in PBS-treated Control-KD1 cells were defined as one-fold and their expression levels in other cell lines/conditions were all compared to those in the PBS-treated Control-KD1 cells. Three independent replicates (n=3 for each replicate) were performed and results represented means of three replicates ± SD. Significant difference was determined by the One-way ANOVA, followed by Tukey's post-hoc test. ns: no significant difference.

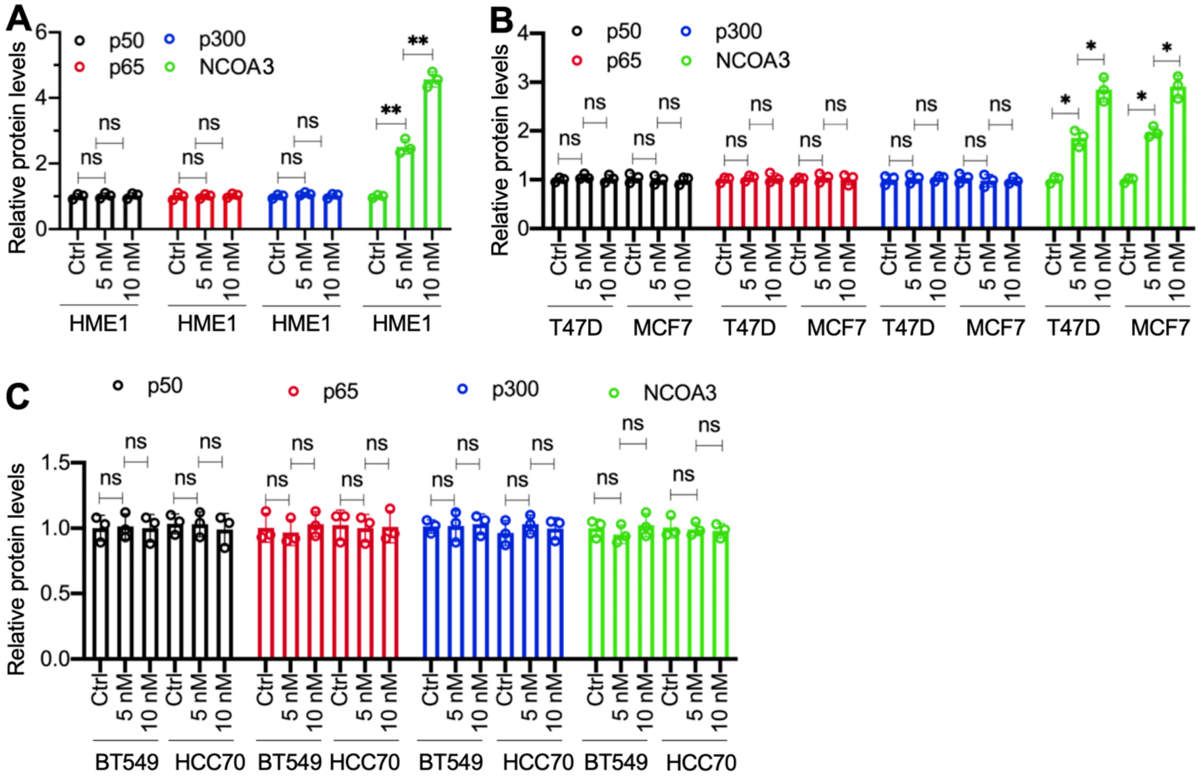

Figure S13. The quantified protein levels of p65, p50, p300 and NCOA3 in HME1, T47D, MCF7, BT549, and HCC70 cells treated with or without E2

Protein signals in Figure 4A-4C were quantified using Image J software and then normalized to their corresponding loading control (GAPDH). Protein levels in PBS-treated HME1 (Ctrl) **(A)**, PBS-treated T47D (Ctrl) **(B)**, and PBS-treated BT549 (Ctrl) **(C)** were defined as one-fold and protein levels in other cells were all compared with those in PBS-treated controls. Results represented means of three replicates ± SD. Significant difference was determined by the One-way ANOVA, followed by Tukey's post-hoc test. ns: no significant difference; * $P < 0.05$ and ** $P < 0.01$.

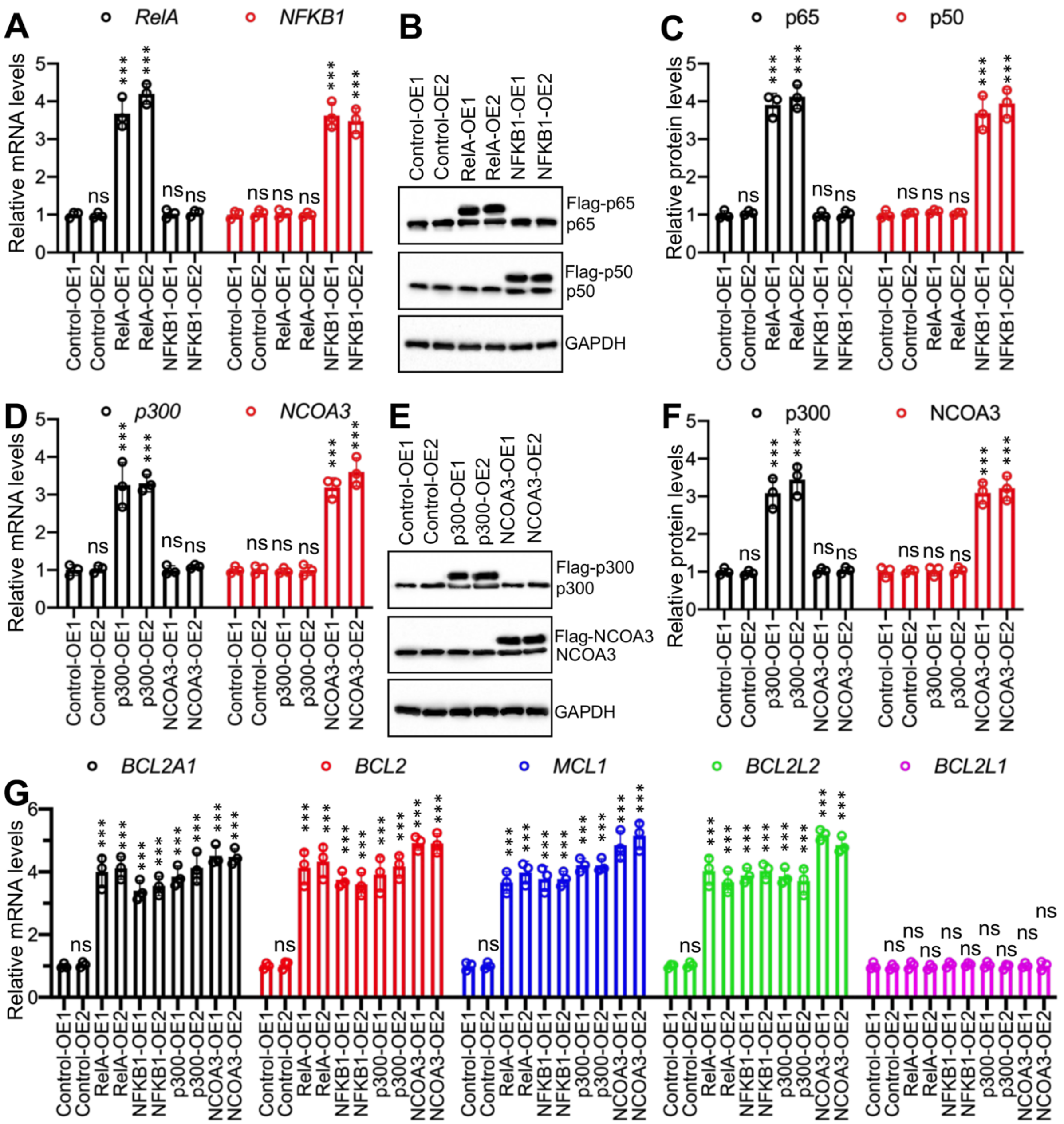

Figure S14. The mRNA and protein levels of RelA, NFKB1, p300 and NCOA3 in their corresponding overexpression cell lines (T47D background)

(A) The mRNA levels of *RelA* and *NFKB1* in Control-OE1/2, RelA-OE1/2 and NFKB1-OE1/2 cells. **(B** and **C)** The protein levels of p65 and p50 in Control-OE1/2, RelA-OE1/2 and NFKB1-OE1/2 cells. **(B)** Western blotting results. **(C)** Quantified protein levels. **(D)** The mRNA levels of *p300* and *NCOA3* in Control-OE1/2, p300-OE1/2 and NCOA3-OE1/2

cells. **(E** and **F)** The protein levels of p65 and p50 in Control-OE1/2, p300-OE1/2 and NCOA3-OE1/2 cells. **(E)** Western blotting results. **(F)** Quantified protein levels. (G) The mRNA levels of *BCL2A1*, *BCL2*, *MCL1*, *BCL2L2*, and *BCL2L1* in Control-OE1/2, RelA-OE1/2, NFKB1-OE1/2, p300-OE1/2, and NCOA3-OE1/2 cells. For the measurement of mRNA levels in this figure, the expression levels of each gene in Control-OE1 cells were defined as one-fold and their expression levels in other cell lines were all compared to those in Control-OE1 cells. Three independent replicates (n=3 for each replicate) were performed and results represented means of three replicates ± SD. Significant difference was determined by the One-way ANOVA, followed by Tukey's post-hoc test. ns: no significant difference; ** $P < 0.01$. For immunoblots, three independent replicates were performed. For each lane in a replicate, three independent protein samples were mixed with equal weights (20 μg for each). One group of immunoblot images were shown. For quantification of protein levels, protein signals were quantified using Image J software and then normalized to their corresponding loading control (GAPDH). Protein levels in Control-OE1 cells were defined as one-fold and protein levels in other cells were all compared with those in Control-OE1. Results represented means of three replicates ± SD. Significant difference was determined by the One-way ANOVA, followed by Tukey's post-hoc test. ns: no significant difference; *** $P < 0.001$.

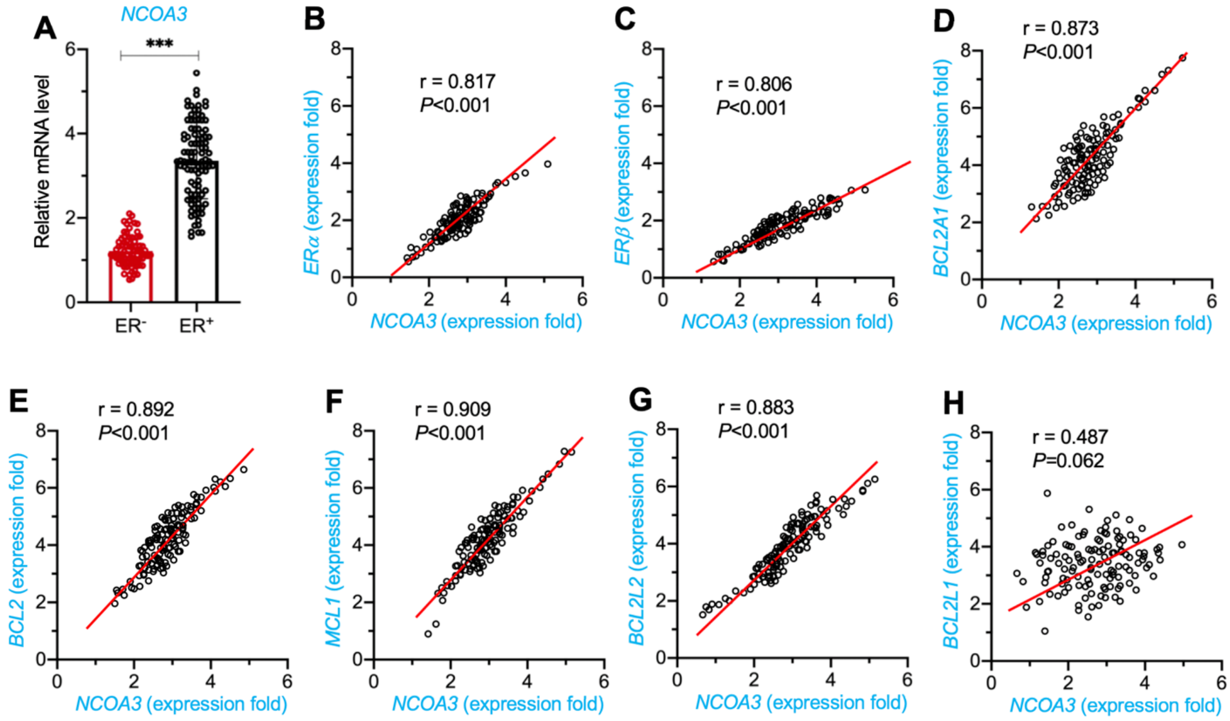

Figure S15. *NCOA3* was overexpressed in ER-positive tumors and it was positively correlated with four antiapoptotic genes

(A) The mRNA level of *NCOA3* in ER-negative and ER-positive tumor samples. The same RNA samples as used in Figure 1D were used for detection of mRNA levels of *NCOA3*. Three independent replicates were performed and one group of representative results was shown. Significant difference was determined by the One-way ANOVA, followed by Tukey's post-hoc test. *** $P < 0.001$. **(B-H)** Results of Pearson's correlation assays. The expression level of *NCOA3* in ER-positive tumor samples (n=102) was used for Pearson's correlation assays with the expression levels of ERα, ERβ, *BCL2A1*, *BCL2*. *MCL1*, *BCL2L2*, and *BCL2L1* with SPSS software. **(B)** Correlation between *NCOA3* and *ERα.* **(C)** Correlation between *NCOA3* and *ERβ.* **(D)** Correlation between *NCOA3* and *BCL2A1.* **(E)** Correlation between *NCOA3* and *BCL2.* **(F)** Correlation between *NCOA3* and *MCL1.* **(G)**

Correlation between *NCOA3* and *BCL2L2.* **(H)** Correlation between *NCOA3* and *BCL2L1.* Three independent replicates were performed and one group of representative results was shown.

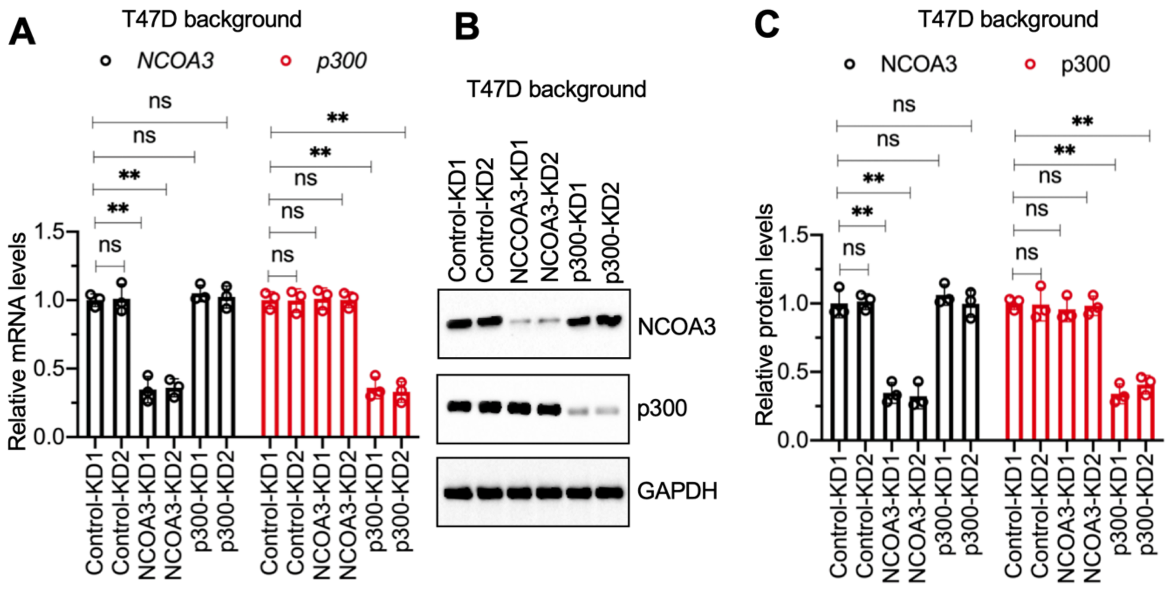

Figure S16. The mRNA and protein levels of p300 and NCOA3 in their corresponding knockdown cell lines under T47D background

(A) The mRNA levels of *NCOA3* and *p300* in Control-KD1/2, NCOA3-KD1/2, and p300-KD1/2 cells (T47D background). The expression levels of *NCOA3* and *p300* in the Control-KD1 cells were defined as one-fold and their expression levels in other cell lines were all compared to that in the Control-KD1 cells. Three independent replicates (n=3 for each replicate) were performed and results represented means of three replicates ± SD. Significant difference was determined by the One-way ANOVA, followed by Tukey's post-hoc test. ns: no significant difference; ** $P < 0.01$. **(B** and **C)** The protein levels of NCOA3 and p300 in Control-KD1/2, NCOA3-KD1/2, and p300-KD1/2 cells (T47D background). **(B)** Western blotting results. Three independent replicates were performed. For each lane in a replicate, three independent protein samples were mixed with equal weights (20 μg for each). One group of immunoblot images were shown. **(C)** Quantified protein levels. protein signals in (B) were quantified using Image J software and then normalized to their

corresponding loading control (GAPDH). Protein levels in Control-KD1 cells were defined as one-fold and protein levels in other cells were all compared with those in Control-KD1. Results represented means of three replicates ± SD. Significant difference was determined by the One-way ANOVA, followed by Tukey's post-hoc test. ns: no significant difference; ** *P* < 0.01.

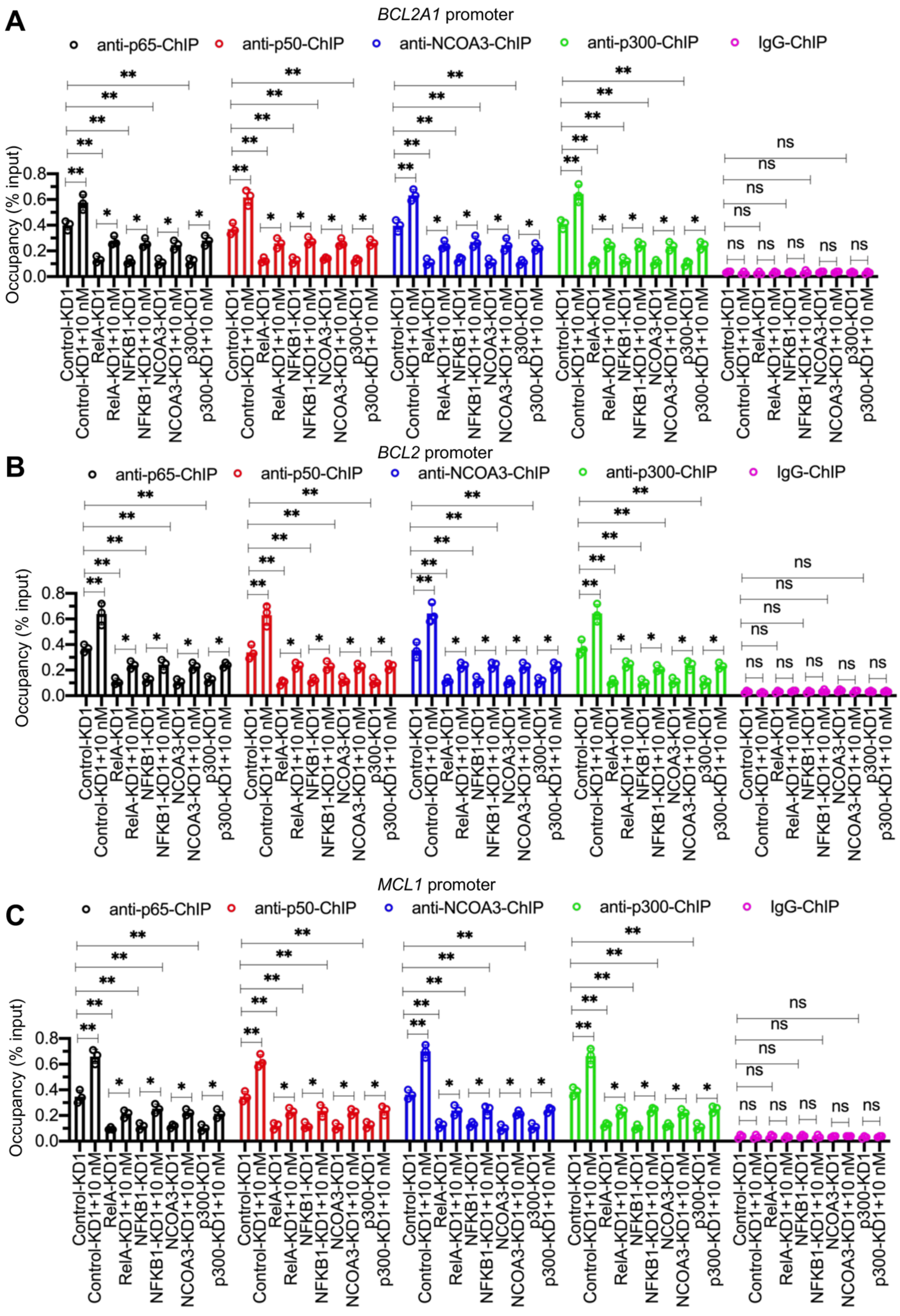

A
BCL2A1 promoter
anti-p65-ChIP
anti-p50-ChIP
anti-NCOA3-ChIP
anti-p300-ChIP
IgG-ChIP
Occupancy (% input)
Control-KD1
Control-KD1+10 nM
RelA-KD1
RelA-KD1+10 nM
NFKB1-KD1
NFKB1-KD1+10 nM
NCOA3-KD1
NCOA3-KD1+10 nM
p300-KD1
p300-KD1+10 nM
B
BCL2 promoter
C
MCL1 promoter

Figure S17. The occupancies of NCOA3-p300-NF-κB members on the promoters of *BCL2A1*, *BCL2* and *MCL1* in NCOA3-p300-NF-κB-KD cells treated with or without E2

The Control-KD1, RelA-KD1, NFKB1-KD1, NCOA3-KD1, and p300-KD1 cells in T47D background were treated with 10 nM E2 or PBS for 4 h. Cells were used for ChIP assays with anti-p65, anti-p50, anti-NCOA3, anti-p300, and IgG-coupled protein G agarose. The input and output DNA samples were used for RT-qPCR analyses to determine the occupancies of NCOA3-p300-NF-κB members on the promoters of *BCL2A1* **(A)**, *BCL2* **(B)**, and *MCL1* **(C)**. Three independent replicates (n=3 for each replicate) were performed and results represented means of three replicates ± SD. Significant difference was determined by the One-way ANOVA, followed by Tukey's post-hoc test. ns: no significant difference; * $P < 0.05$ and ** $P < 0.01$.

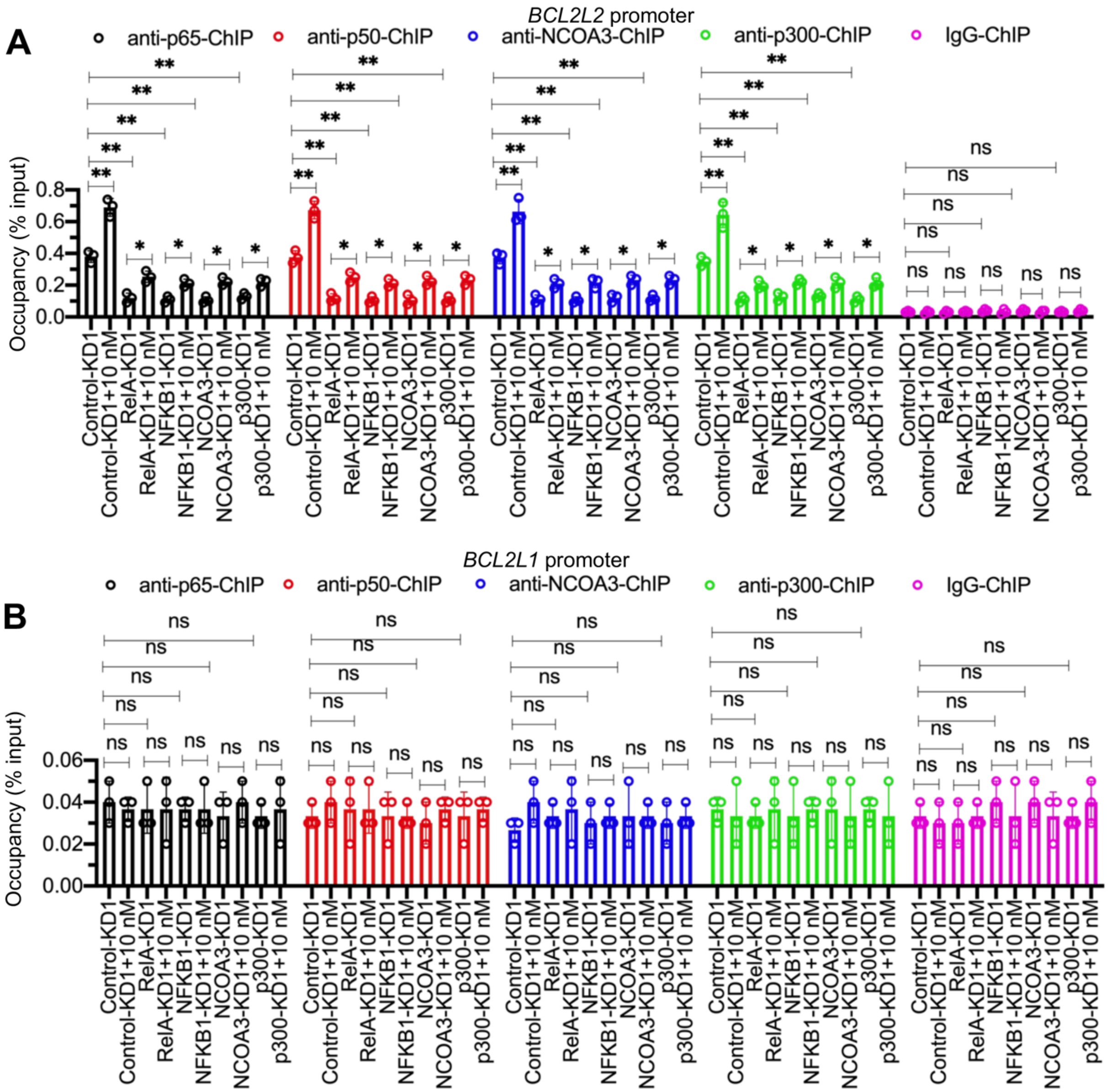

Figure S18. The occupancies of NCOA3-p300-NF-κB members on the promoters of *BCL2L1* and *BCL2L2* in NCOA3-p300-NF-κB-KD cells treated with or without E2

The same input and output DNA samples as in Figure S17 were used for RT-qPCR analyses to determine the occupancies of NCOA3-p300-NF-κB members on the promoters of *BCL2L2* **(A)** and *BCL2L1* **(B)**. Three independent replicates (n=3 for each replicate) were performed and results represented means of three replicates ± SD.

Significant difference was determined by the One-way ANOVA, followed by Tukey's post-hoc test. ns: no significant difference; * $P < 0.05$ and ** $P < 0.01$.

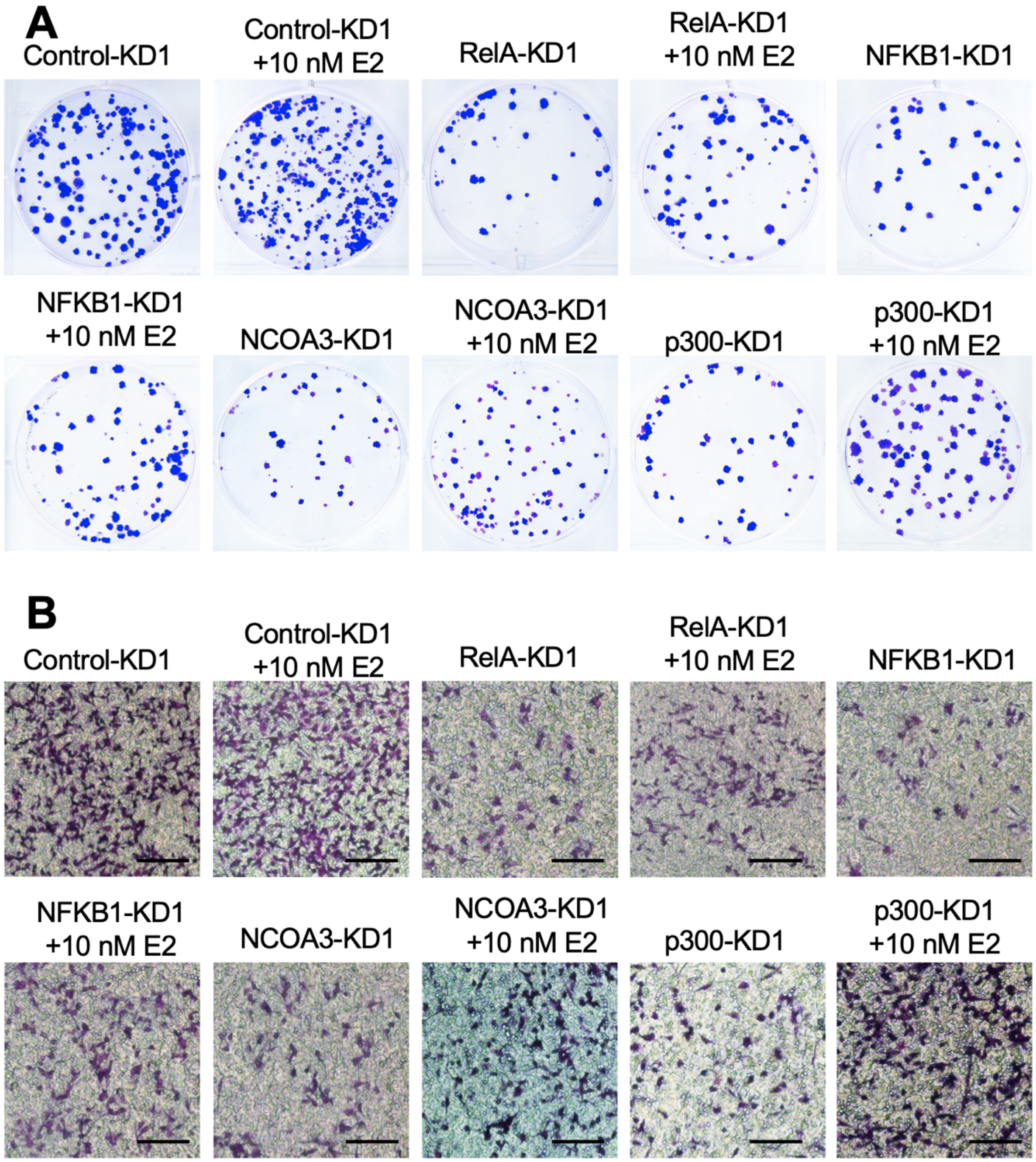

Figure S19. Deficiency of NCOA3-p300-NF-κB members inhibited breast cancer cell proliferation and invasion

(A) Representative images of colony formation. The Control-KD1, p65-KD1, p50-KD1, p300-KD1, and NCOA3-KD1 cells in T47D background were seeded into 6-well plates at

a density of 1 $\times$ 10^3 cells/well in 2 mL DMEM containing 10% charcoal stripped FBS and 10 nM E2 (or PBS for control). Cell colonies were fixed with 5% glutaraldehyde at 37°C for 10 min, followed by staining with 0.1% crystal violet. Three independent replicates (n=3 for each replicate) were performed and one group of images were shown. **(B)** Representative images of cell invasion. The same cells as in (A) were seeded in the upper compartment of the Nunc Polycarbonate Cell Culture Inserts at a density of 1 $\times$ 10^5 cells/well in the conditions of 10 nM E2 and PBS (control). After incubation at 37°C for 48 h, invaded cells in the lower insert were fixed with 5% glutaraldehyde and stained with 0.1% crystal violet. Three independent replicates (n=3 for each replicate) were performed and one group of images were shown. Bars=50 μm.

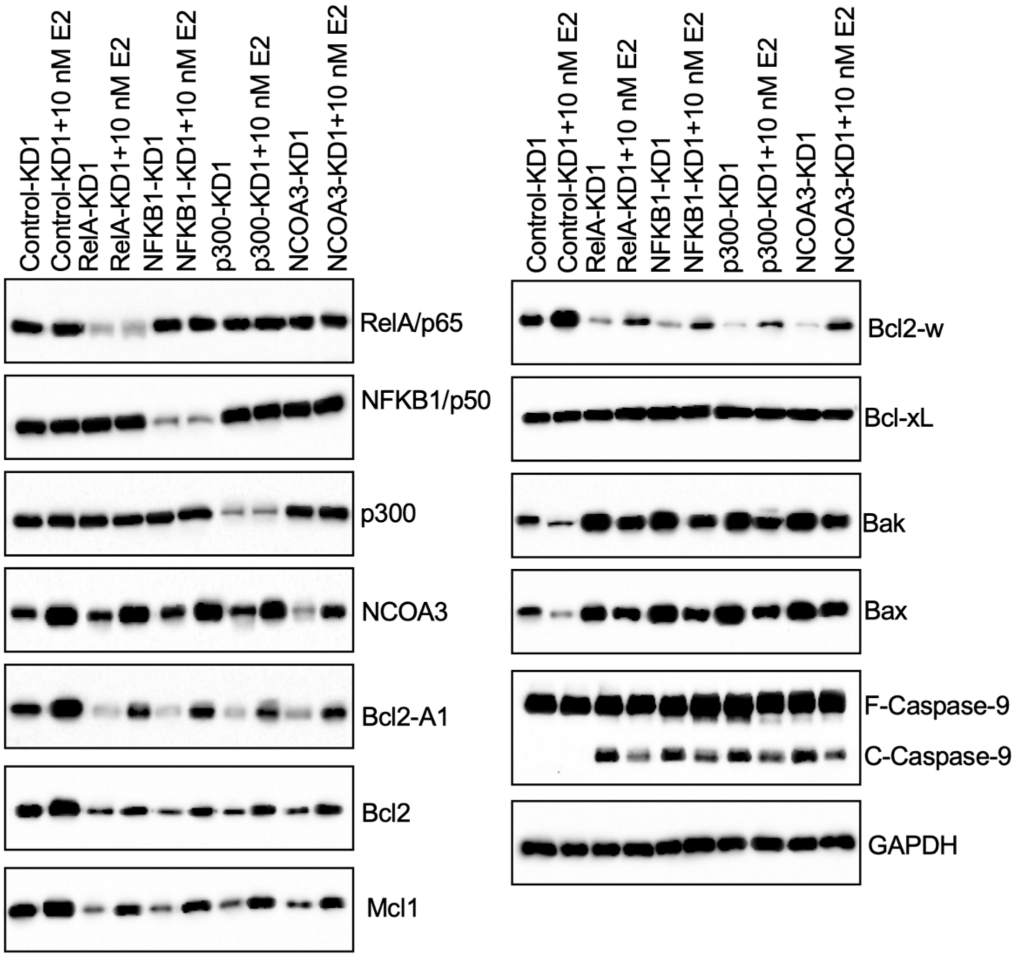

Figure S20. The protein levels of NCOA3-p300-NF-κB members, five antiapoptotic proteins and three apoptotic maker proteins in NCOA3-p300-NF-κB-KD cells treated with or without E2

The Control-KD1, RelA-KD1, NFKB1-KD1, NCOA3-KD1, and p300-KD1 cells in T47D background were treated with 10 nM E2 or PBS for 4 h, followed by protein isolation and immunoblot assays to examine protein levels of p65, p50, NCOA3, p300, Bcl2A1, Bcl2, Mcl1, Bcl-w, Bcl-xL Bak, Bax, Caspase-9 (F: full length; C: cleaved length), and GAPDH (loading control). Three independent replicates were performed. For each lane in a

replicate, three independent protein samples were mixed with equal weights (20 μg for each). One group of immunoblot images were shown.

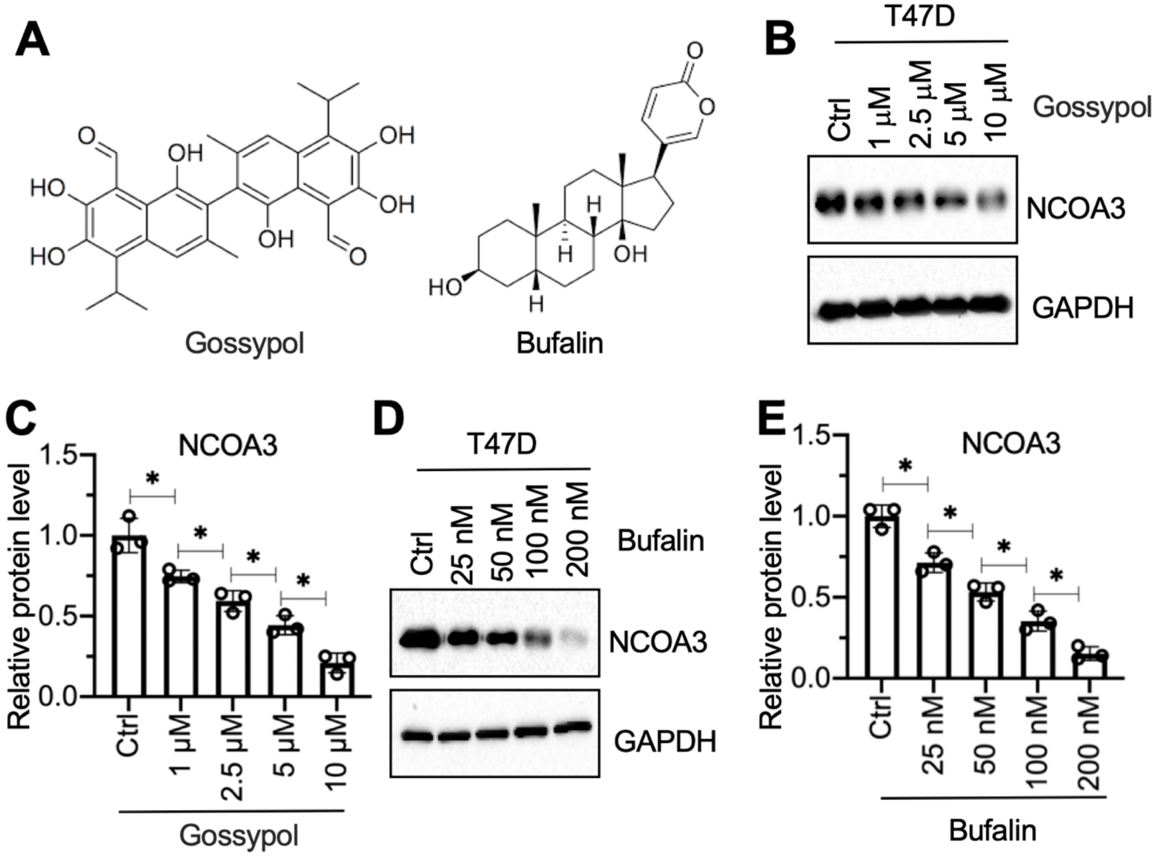

Figure S21. Gossypol and bufalin dose-dependently inhibited NCOA3 protein level

(A) The chemical structures of two NCOA3 inhibitors (gossypol and bufalin). **(B** and **C)** NCOA3 protein level in gossypol-treated cells. T47D cells were treated with PBS (Ctrl), 1, 2.5, 5 and 10 μM gossypol for 6 h, followed by examination of NCOA3 and GAPDH protein levels. **(B)** Immunoblot results. **(C)** Quantified protein levels. **(D** and **E)** NCOA3 protein level in bufalin-treated cells. T47D cells were treated with PBS (Ctrl), 25, 50, 100 and 200 nM bufalin for 6 h, followed by examination of NCOA3 and GAPDH protein levels. **(D)** Immunoblot results. **(E)** Quantified protein levels. For immunoblots, three independent replicates were performed. For each lane in a replicate, three independent protein samples were mixed with equal weights (20 μg for each). One group of immunoblot

images were shown. For quantification of protein levels, protein signals were quantified using Image J software and then normalized to their corresponding loading control (GAPDH). Protein levels in PBS-treated T47D cells were defined as one-fold and protein levels in other cells were all compared with those in PBS-treated T47D cells. Results represented means of three replicates ± SD. Significant difference was determined by the One-way ANOVA, followed by Tukey's post-hoc test. * $P < 0.05$.

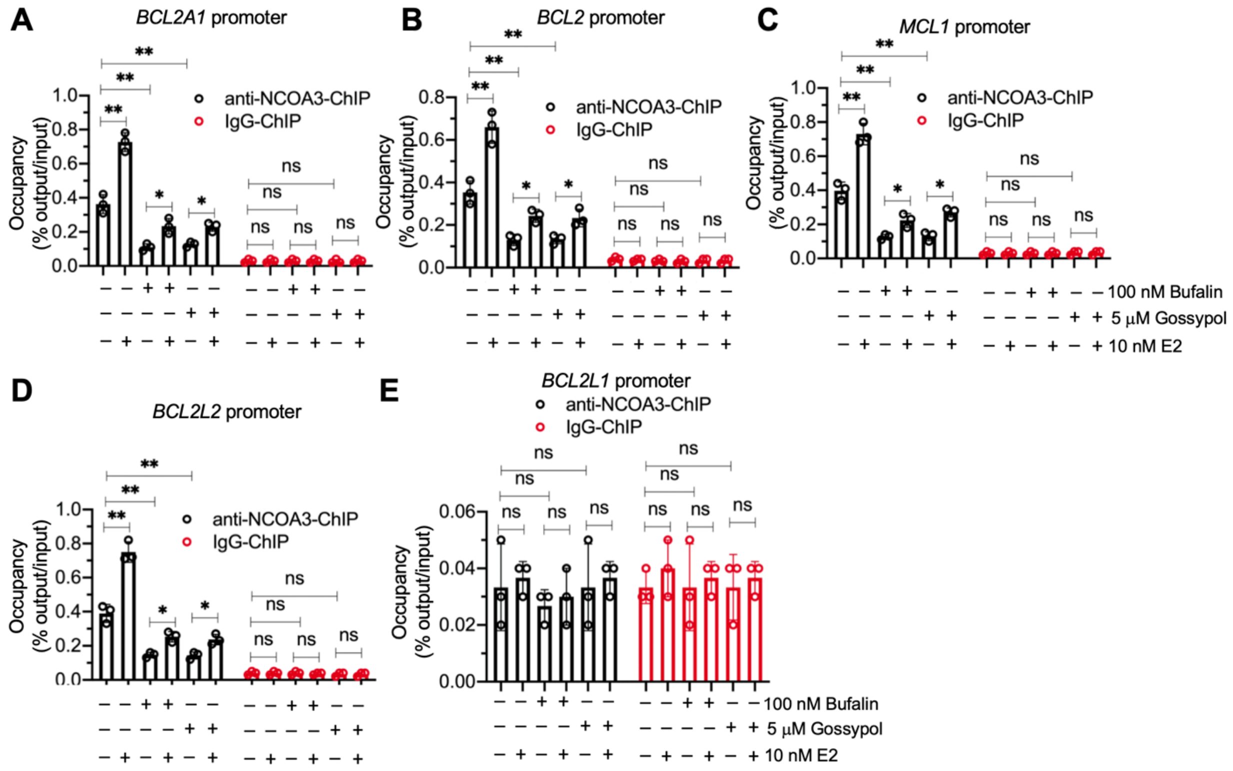

Figure S22. Treatments with gossypol and bufalin decreased the occupancy of NCOA3 on the promoters of *BCL2A1*, *BCL2* and *MCL1* T47D cells treated with or without E2

The T47D cells were treated with PBS, 5 μM gossypol and 100 nM bufalin for 6 h, followed by treatment with 10 nM E2 or PBS for 4 h. Cells were used for ChIP assays with anti-NCOA3 and IgG-coupled protein G agarose. The input and output DNA samples were used for RT-qPCR analyses to determine the occupancy of NCOA3 on the promoters of *BCL2A1* **(A)**, *BCL2* **(B)**, *MCL1* **(C)**, *BCL2L2* **(D)**, and *BCL1L1* **(E)**. Three independent replicates (n=3 for each replicate) were performed and results represented means of three replicates ± SD. Significant difference was determined by the One-way ANOVA, followed by Tukey's post-hoc test. ns: no significant difference; * $P < 0.05$ and ** $P < 0.01$.

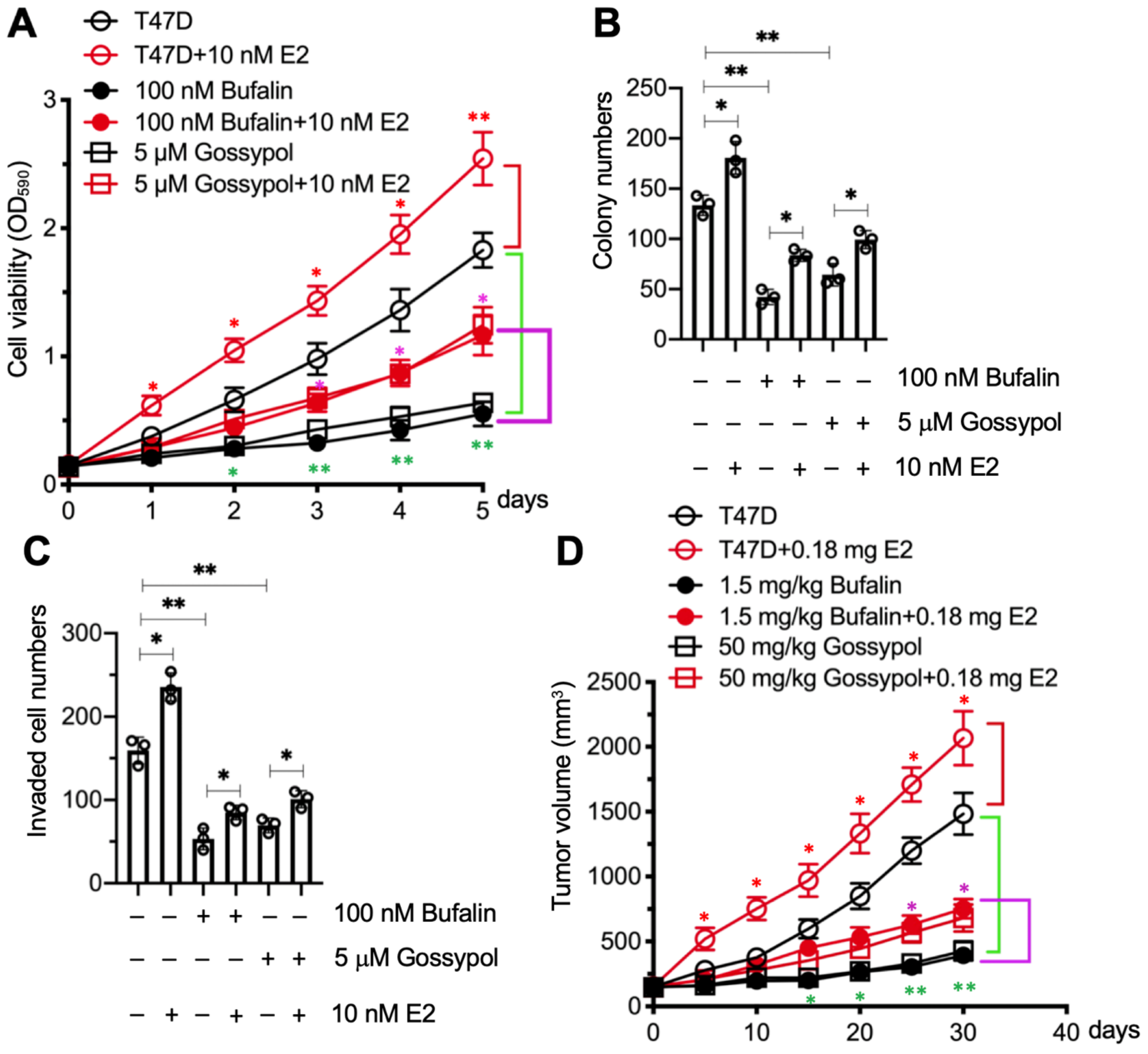

Figure S23. Gossypol and bufalin inhibited breast cancer cell proliferation, invasion, and in vivo tumor growth

(A) Cell viability. The T47D cells were used for MTT assay in the conditions of PBS, PBS+10 nM E2, 5 μM gossypol, 5 μM gossypol +10 nM E2, 100 nM bufalin, and 100 nM bufalin+10 nM E2 at different time points (0, 1, 2, 3, 4, and 5 days). Three independent replicates (n=3 for each replicate) were performed and results represented means of three replicates ± SD. Significant difference was determined by the One-way ANOVA,

followed by Tukey's post-hoc test. At each time point, comparisons of T47D *vs.* T47D+10 nM E2 (red asterisks); T47D *vs.* 100 nM bufalin; T47D *vs.* 5 μM gossypol; 100 nM bufalin *vs.* 100 nM bufalin+10 nM E2; and 5 μM gossypol *vs.* 5 μM gossypol+10 nM E2. The statistical differences between T47D *vs.* 100 nM bufalin and T47D *vs.* 5 μM gossypol were similar. Only one set of statistical difference (green asterisks) is shown due to the overlapped curves. The statistical differences between 100 nM bufalin *vs.* 100 nM bufalin+10 nM E2 and 5 μM gossypol *vs.* 5 μM gossypol+10 nM E2 were similar. Only one set of statistical difference (purple asterisks) is shown due to the overlapped curves. * $P < 0.05$ and ** $P < 0.01$. **(B)** Colony numbers. Cells in (A) were used for colony formation assay. **(C)** Invaded cell numbers. Cells in (A) were used for cell invasion assay. For experiments in (B) and (C), three independent replicates (n=3 for each replicate) were performed and results represented means of three replicates ± SD. Significant difference was determined by the One-way ANOVA, followed by Tukey's post-hoc test. * $P < 0.05$. **(D)** Tumor volumes. T47D cells were injected into female nude mice (n=60 for each cell line), followed by implantation with or without 0.18 mg E2 pellet. Mice were randomly grouped into three groups (Control, gossypol, and bufalin) when tumor volumes reached ~150 mm^3. The Control group mice were injected with PBS. The gossypol groups of mice were injected with 50 mg/kg gossypol and the bufalin groups of mice were injected with 1.5 mg/kg bufalin. Tumor volumes were measured at 5-day intervals for 30 days. Three independent replicates (n=10 for each replicate) were performed and results represented means of three replicates ± SD. Significant difference was determined by the One-way ANOVA, followed by Tukey's post-hoc test. At each time point, comparisons of T47D *vs.* T47D+0.18 mg E2 (red asterisks); T47D *vs.* 1.5 mg/kg bufalin; T47D *vs.* 50 mg/kg

gossypol; 1.5 mg/kg bufalin *vs.* 1.5 mg/kg bufalin+0.18 mg E2; and 50 mg/kg gossypol *vs.* 50 mg/kg gossypol+0.18 mg E2 were performed. The statistical differences between T47D *vs.* 1.5 mg/kg bufalin; T47D *vs.* 50 mg/kg gossypol were similar. Only one set of statistical difference (green asterisks) is shown due to the overlapped curves. The statistical differences between 1.5 mg/kg bufalin *vs.* 1.5 mg/kg bufalin+0.18 mg E2; and 50 mg/kg gossypol *vs.* 50 mg/kg gossypol+0.18 mg E2 were similar. Only one set of statistical difference (purple asterisks) is shown due to the overlapped curves. * $P < 0.05$ and ** $P < 0.01$.

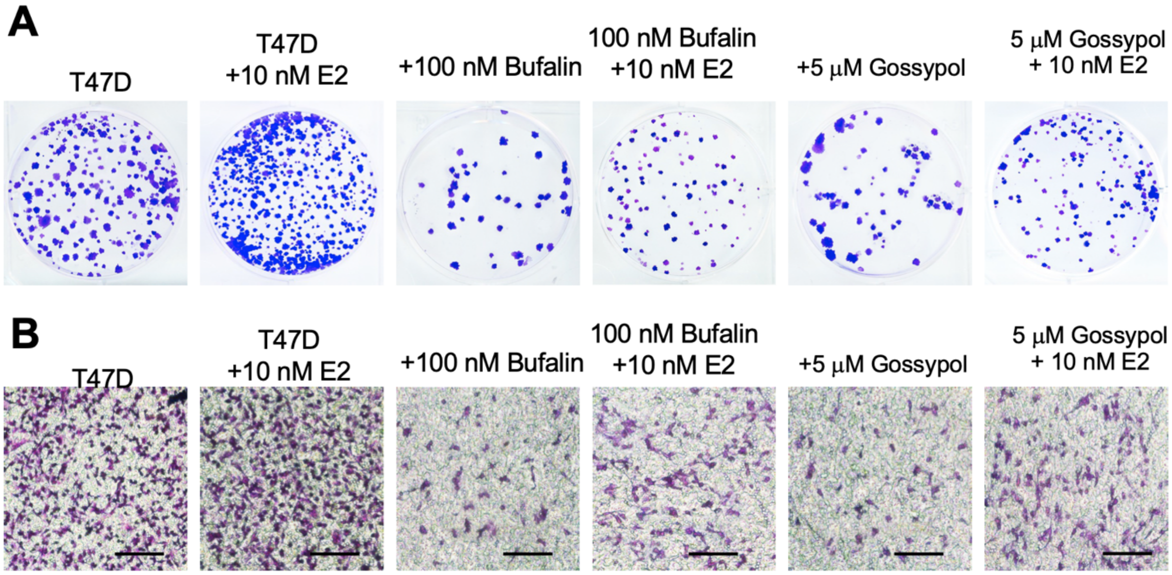

Figure S24. Gossypol and Bufalin inhibited breast cancer cell proliferation and invasion

(A) Representative images of colony formation. T47D cells were grown in the conditions of PBS, PBS+10 nM E2, 5 μM gossypol, 5 μM gossypol +10 nM E2, 100 nM bufalin, and 100 nM bufalin+10 nM E2 at a density of 1×10^3 cells/well in 2 mL DMEM containing 10% charcoal stripped FBS. Cell colonies were fixed with 5% glutaraldehyde at 37°C for 10 min, followed by staining with 0.1% crystal violet. Three independent replicates (n=3 for each replicate) were performed and one group of images were shown. **(B)** Representative images of cell invasion. The same cells as in (A) were seeded in the upper compartment of the Nunc Polycarbonate Cell Culture Inserts at a density of 1×10^5 cells/well in the conditions of 10 nM E2 and PBS (control). After incubation at 37°C for 48 h, invaded cells in the lower insert were fixed with 5% glutaraldehyde and stained with 0.1% crystal violet. Three independent replicates (n=3 for each replicate) were performed and one group of images were shown. Bars=50 μm.
