# Supplementary material for: Estrogen-dependent activation of NCOA3 couples with p300 and NF-κB to mediate antiapoptotic genes in ER-positive breast cancer cells
Source: Discov Oncol. 2023 Feb 28;14:28. doi: 10.1007/s12672-023-00635-0 (PMC9975134; doi:10.1007/s12672-023-00635-0)
Supplement: Supplementary file 2 — Supplementary file2 (DOCX 27 KB) [file 12672_2023_635_MOESM2_ESM.docx]

**Table S1. The basic information of ER-negative and ER-positive patients**

| **Patients** | **Tumor stages** | **Numbers (n)** | **Average ages** | **Diagnosis method** |
| --- | --- | --- | --- | --- |
| ER-negative | I | 20 | 54.6±6.1 | IHC and EGD |
|  | II | 23 | 57.2±6.7 | IHC and EGD |
|  | III | 30 | 60.4±7.2 | IHC and EGD |
| ER-positive | I | 31 | 53.3±5.8 | IHC and EGD |
|  | II | 39 | 56.5±6.5 | IHC and EGD |
|  | III | 32 | 62.6±7.8 | IHC and EGD |

IHC: Immunohistochemistry; EGD: ER gene expression detection

**Table S2. Vectors, insertion sites, and primer sequences**

| **Vectors** | **Insertion sites** | **Forward oligoes** | **Reverse oligoes** |
| --- | --- | --- | --- |
| pcDNA3-Flag-p65 | EcoRI + XhoI | CGGAATTCATGGACGAACTGTTCCCCCTCAT | CCGCTCGAGTCTTAGGAGCTGATCTGACTCAGC |
| pcDNA3-Flag-p50 | EcoRI + XhoI | CGGAATTCATGGCAGAAGATGATCCATATTTGG | CCGCTCGAGCTAAATTTTGCCTTCTAGAGGTCCT |
| pcDNA3-Flag-p300 | EcoRI + XhoI | CGGAATTCATGGCCGAGAATGTGGTGGAACCG | CCGCTCGAGCTAGTGTATGTCTAGTGTACTCTGTG |
| pcDNA3-Flag-NCOA3 | EcoRI + XhoI | CGGAATTCATGAGTGGATTAGGAGAAAACTTG | CGGAATTCTCAGCAGTATTTCTGATCAGGACCCATAG |
| pcDNA3-Myc-p50 | BamHI + NotI | CGGGATCCATGGCAGAAGATGATCCATATTTGG | ATAAGAATGCGGCCGCCTAAATTTTGCCTTCTAGAGGTCCT |
| pcDNA3-Myc-p300 | BamHI + NotI | CGGGATCCATGGCCGAGAATGTGGTGGAACCG | ATAAGAATGCGGCCGCCTAGTGTATGTCTAGTGTACTCTGTG |
| pcDNA3-Myc-p65 | BamHI + NotI | CGGGATCCATGGACGAACTGTTCCCCCTCAT | ATAAGAATGCGGCCGCTTAGGAGCTGATCTGACTCAGC |

**Table S3. shRNA sequences and order information**

| **Genes** | **shRNA target sequences** | **Clone IDs** | **Sources** |
| --- | --- | --- | --- |
| RelA | GCCTTAATAGTAGGGTAAGTT | TRCN0000014683 | Sigma-Aldrich |
|  | CGGATTGAGGAGAAACGTAAA | TRCN0000014684 | Sigma-Aldrich |
| NFKB1 | CCAGAGTTTACATCTGATGAT | TRCN0000006518 | Sigma-Aldrich |
|  | CGAATGACAGAGGCGTGTATA | TRCN0000006521 | Sigma-Aldrich |
| p300 | GCCTTCACAATTCCGAGACAT | TRCN0000039885 | Sigma-Aldrich |
|  | CCCGGTGAACTCTCCTATAAT | TRCN0000039886 | Sigma-Aldrich |
| NCOA3 | CCTCTACATCTGGAGGAGTAT | TRCN0000019702 | Sigma-Aldrich |
|  | GCAGTCTATTCGTCCTCCATA | TRCN0000019703 | Sigma-Aldrich |

**Table S4. Gene primers for RT-qPCR analyses**

| **Genes** | **Forward oligoes** | **Reverse oligoes** |
| --- | --- | --- |
| BCL2 | CATTATAAGCTGTCGCAGA | ATGGGGCGTGTGCCCGGGCTG |
| BCL2A1 | TGAAGTCATGCTTGGACAATG | CAGTTAATGATGCCGTCTTCA |
| BCL2L1 | CTTCAACCGCTGGTTCCTGA | GGCAATGGCGGCTGGACGGA |
| BCL2L2 | ATGAGTTCGAGACCCGCTTC | AAGGCTACAAGGCGGCCCCAG |
| MCL1 | CCACGAGACGGCCTTCCAAG | ATGAGAGTCACAATCCTGC |
| RELA | CAGACCCAGCTGTGTTCACA | TAGCCTCAGGGTACTCCAT |
| NFKB1 | ATCTACTAGAAGTCACATCTG | AGCGGTCCAGAAGGCTCAGGTC |
| P300 | TGGAGTTCTCTCCACAGACA | TTGGATCTCATTGAAACACT |
| NCOA3 | TCTGTGCAGTCTATTCGTCC | CTATCCATCATTCTTGGATTCC |
| β-Actin | CACCATTGGCAATGAGCGGTTC | AGGTCTTTGCGGATGTCCACGT |

**Table S5. Primers for ChIP assays**

| **Gene promoters** | **Forward oligoes** | **Reverse oligoes** |
| --- | --- | --- |
| BCL2 | TCACAGCAGGGCAGCGCT | CGTGTATGAGAGTGTGTACA |
| BCL2A1 | AGTGCTAGGATTACAGGC | GAGGAAGGAGTAACTGCT |
| BCL2L1 | CAGGACAATCACTTGAACCT | GTTCGAGATGGAGTCTTGCT |
| BCL2L2 | AGGCGCCTCGTCTCAGATA | GGCCGTGACTATGCTCTA |
| MCL1 | CAACAGAGCTAGACTGTC | ATCTCTGTTCCATAGTCTTT |

**Table S6. Identification of p65-interacting proteins in T47D cells by MS analysis**

| **Proteins** | **Protein description** | **Percolator score** | **Molecular weight (kD)** | **Matched queries** | **Matched peptides** |
| --- | --- | --- | --- | --- | --- |
| p65 | Nuclear Factor NF-Kappa-B P65 Subunit | 4787 | 60 | 32 | 20 |
| p50 | Nuclear Factor NF-Kappa-B P50 Subunit | 4369 | 105 | 20 | 15 |
| p300 | Histone Acetyltransferase P300 | 4228 | 264 | 65 | 33 |
| NCOA3 | Nuclear Receptor Coactivator 3 | 4197 | 155 | 33 | 20 |
| POLD3 | DNA Polymerase Delta 3, Accessory Subunit | 3877 | 51 | 19 | 4 |
| NR3C2 | Nuclear Receptor Subfamily 3 Group C Member 2 | 3514 | 107 | 20 | 10 |
| ANXA4 | Annexin A4 | 3211 | 36 | 33 | 19 |
| NRG1 | Neuregulin 1 | 3029 | 70 | 25 | 13 |
| JAK2 | Janus Kinase 2 | 2877 | 131 | 20 | 14 |
| NFATC1 | Nuclear Factor Of Activated T Cells 1 | 2845 | 101 | 23 | 20 |
| NFKB1B | NFKB Inhibitor Beta | 2778 | 38 | 15 | 10 |
| DCTN1 | Dynactin Subunit 1 | 2706 | 142 | 40 | 27 |
| SYT1 | Synaptotagmin 1 | 2633 | 48 | 30 | 12 |
| CNTN2 | Contactin 2 | 2573 | 113 | 28 | 9 |
| UBC | Ubiquitin C | 2449 | 77 | 20 | 14 |
| KPNB1 | Karyopherin Subunit Beta 1 | 2336 | 97 | 15 | 9 |
| KISS1 | KiSS-1 Metastasis Suppressor | 2208 | 15 | 5 | 4 |
| ABCB1 | ATP Binding Cassette Subfamily B Member 1 | 2099 | 141 | 25 | 20 |
| SYT2 | Synaptotagmin 2 | 2011 | 47 | 20 | 10 |
| RPS6KA1 | Ribosomal Protein S6 Kinase A1 | 1987 | 83 | 29 | 18 |
| CDX2 | Caudal Type Homeobox 2 | 1925 | 34 | 11 | 9 |
| SUMO1 | Small Ubiquitin Like Modifier 1 | 1882 | 12 | 10 | 10 |
| H4C1 | H4 Clustered Histone 1 | 1803 | 11 | 5 | 5 |
| TTC5 | Tetratricopeptide Repeat Domain 5 | 1774 | 49 | 17 | 12 |
| MCRS1 | Microspherule Protein 1 | 1536 | 52 | 33 | 13 |
| NAP1L1 | Nucleosome Assembly Protein 1 Like 1 | 1522 | 45 | 20 | 10 |
| USP1 | Ubiquitin Specific Peptidase 1 | 1472 | 88 | 21 | 20 |
| RBM25 | RNA Binding Motif Protein 25 | 1444 | 100 | 33 | 13 |
| CBX1 | Chromobox 1 | 1339 | 21 | 6 | 4 |
| SOX9 | SRY-Box Transcription Factor 9 | 1302 | 56 | 22 | 11 |
| PRMT1 | Protein Arginine Methyltransferase 1 | 1190 | 42 | 17 | 14 |
| CCNT1 | Cyclin T1 | 1172 | 81 | 36 | 13 |
| Eno1 | Enolase 1 | 1086 | 47 | 31 | 10 |
| POLD4 | DNA Polymerase Delta 4 | 1054 | 12 | 4 | 3 |
| NR5A1 | Nuclear Receptor Subfamily 5 Group A Member 1 | 1022 | 52 | 32 | 20 |
| HAT1 | Histone Acetyltransferase 1 | 955 | 50 | 18 | 10 |
| NPM1 | Nucleophosmin 1 | 912 | 33 | 17 | 13 |
| MYOD1 | Myogenic Differentiation 1 | 889 | 35 | 10 | 10 |
| VHL | Von Hippel-Lindau Tumor Suppressor | 843 | 24 | 8 | 7 |
| PREX1 | Phosphatidylinositol 3,4,5-Trisphosphate-Dependent Rac Exchanger 1 Protein | 733 | 186 | 33 | 14 |
| CARM1 | Coactivator Associated Arginine Methyltransferase 1 | 701 | 66 | 20 | 13 |
| DDX27 | DEAD-Box Helicase 27 | 665 | 90 | 30 | 20 |
| SKP2 | S-Phase Kinase Associated Protein 2 | 604 | 48 | 11 | 9 |
| ATAD2 | ATPase Family AAA Domain Containing 2 | 601 | 159 | 40 | 19 |
| NUDT21 | Nudix Hydrolase 21 | 552 | 26 | 13 | 9 |
